# Analectic

**Published:** 1833

**Authors:** 


### Miscellaneous Intelligence.

### ANALECTIC, ANALYTICAL, AND ORIGINAL.

### I. Analectic.

1. *Professor Ehrenberg's discoveries relative to the Structure and Functions of the Infusoria.*—If the obscurity which involves many of the most interesting phenomena in human physiology be ever dispelled, it can only be by the lights furnished by comparative anatomy, and we therefore conceive that we cannot occupy a few pages better than in devoting them to an account of the discoveries of Professor Ehrenberg, of Berlin, relative to the structure and functions of the Infusoria. These discoveries constitute an epoch in that department of science, both from the capital discoveries already made as well as from the direction they give to future researches. The best account we have seen of these discoveries, is that by Dr. Gairdner, in the *Edinburgh New Philosophical Journal*, for September, 1831, and we shall make free use of it in making up the following sketch of the structure and functions of the Infusoria.

Our readers will perhaps be surprised at the mention of the structure and functions of animals, the discovery of whose mere existence has been until recently deemed the ultimum of zoological research, and regarding whom, the sum total of our knowledge has been hitherto confined to a few details on their external forms and active motions. Yet, in the midst of their transparent tissues, the German naturalist has, by a peculiarly ingenious method of observation, developed a highly complicated organization, which with those who arrange the animal kingdom in a linear series, will remove them far from the extremity of the scale. The existence of a digestive, muscular, and generative apparatus, is established beyond doubt; and organs have been also discovered, which bear great analogy with the vascular and nervous systems.

Before entering into the detail of the organization of the infusoria, it is proper to state briefly the method by which the organs are rendered visible. This consists in furnishing the infusoria with organic coloring matter for nutriment. Simple as this may appear, it was not until after ten year's observations, that Dr. Ehrenberg succeeded in selecting the fittest substances, and in applying them in the manner best adapted for the satisfactory exhibition of the phenomena. It was not until he used pure indigo, that these experiments succeeded in a desirable manner. Immediately on a minute particle of a highly attenuated solution of this substance being applied to a drop of water containing some of the pedunculated vorticella, (which are best adapted for the first observation,) and placed under the object glass of the microscope, the most beautiful phenomena presents itself to the eye.—Currents are excited in all directions by the rapid motion of the cilæ, which forms the crown round the anterior part of the animalcule's body, and indicated by the movements of the particles of indigo in a state of very minute division in different directions, and generally all converging towards the orifice or mouth of the animal, situated not in the centre of the crown of cilæ, but between the two rows of these organs which exist concentric to one another. The attention is no sooner excited by this most singular and beautiful phenomenon, when presently the body of the animal, which had been quite transparent, and bearing much resemblance in aspect to some of the marine Rhizostomæ, becomes dotted with a number of distinctly circumscribed circular spots, of a dark blue color, exactly corresponding to that of the moving particles of indigo. In some species, particularly those which are provided with an annular contraction or neck, (such as the *Rotifer vulgaris,*) separating the head from the body, the indigo particles can be traced in a continuous line in their progress from the mouth to these internal cavities.

It is requisite in these experiments to employ coloring matter which does not chemically combine with water, but is only diffused in a state of very minute division. Indigo, carmine, and sap green, are three substances which answer very well the necessary conditions, and are easily recognised by the microscope. But whatever substance is used we must be very particular that it contains no lead, and impurity which very frequently enters into the colors of commerce.

The microscope which Dr. Ehrenberg has used in all his investigaions is one constructed by Chevalier of Paris; it possesses a power of 800. In very few cases, however, is it necessary to use the higher power, and only to demonstrate the existence of an internal cavity in those species which do not exceed from 1-1500th to 1-2000th of a line in diameter, such as the *Monas termo*, *atomas*, and *lens*, and which almost elude the power even of so powerful an instrument. In almost all cases, a power from 300 to 400 is sufficient; and Dr. Ehrenberg has made all his observations and drawings of the structure of the *Hydatina senta* with a power of 380.

In conformity with the great axiom of scientific observation, to measure every thing which is capable of measurement, Dr. Ehrenberg has not neglected to express in numbers the dimensions not only of the totality, but also of the integrant parts of these beings, placed as it were at the verge of organised nature. For this purpose he uses a glass micrometer, constructed by Dolland, which gives directly the ten-thousandth part of an inch, and permits of a much smaller quantity being correctly estimated, as it contains the astonishing number of 400 equal parts distinctly cut in glass within the space of half a line. By means of a micrometer screw, which has been since constructed by Pistor of Berlin, he has been enabled to measure directly 1-48000th of an inch, or 1-4000th of a line, a degree of minuteness which is never necessary in actual practice.

1. *Digestive System.*—By the use of coloring matter in the way above-mentioned, a digestive system has been demonstrated in *all* the genera of this class of animals, distinctly characterised by Muller.—This fact Dr. Ehrenberg states in the following proposition: "All true infusoria, even the smallest monads, are not a homogeneous jelly, but organised animal bodies, distinctly provided with at least a mouth and internal nutritive apparatus." In none has the cuticular absorption of nutritive matter ever been observed, which had been the opinion of all previous writers upon the subject, not from any positive observations, but merely from their inability otherwise to explain the nutrition of these animals. Generations of these transparent gelatinous bodies may remain immersed for weeks in an indigo solution, without presenting any colored points in their tissue, except the circumscribed cavities above referred to; and when in a state of activity, the minute particles of indigo and carmine are seen to hurry rapidly over the whole surface of their transparent bodies, in order to reach the mouth, generally situate at one or other of their extremities. Indeed there is no necessity of having recourse to such a supposition, when we can clearly see the prehension of coloring particles, their reception into a mouth, and conveyance from thence into an internal stomach or stomachs.

The alimentary canal presents, as in other classes of the animal kingdom, the utmost variety in respect to form, situation and degree of complication. It is in the *Monas termo pulvisculus*, and other larger monads, simply a round sac in the centre, and occupying the greater part of their bodies. In the genera *Enchelys Paramœcium* and *Kolpoda*, it assumes the form of a long intestinal canal, traversing the greater part of the body, and at times convoluted in a spiral manner, which is furnished with a great number of cæcal appendages, or stomachs; this singular disposition, of which no other example occurs in the animal kingdom, is particularly distinct in the *Leucophrys patula*. That these blind sacs are real stomachs, and do not at all correspond to the cæca of other animals, is evident from the fact of their being filled with coloring matter immediately on its being received at the mouth, or anterior orifice of the canal. The tubes which connect these sacs to the main canal of the intestine, vary very much, both in length and in diameter, as well among the different cæca, as in the same one at different times, being usually in a state of great contraction, and at times scarcely perceptible when the cavity to which it belongs is empty, and may be supposed not to be in a state of activity.—We can count from one hundred to two hundred of these sacs in the course of the intestines of the *Paramœcium chrysalis* and *aurela.*—When they are filled with coloring matter, the common intestinal tube is usually quite empty and transparent, this, joined to the bluish, reddish or greenish tint which they often assume when empty, may have been the reason that these sacs were mistaken by Muller for ova, and by Schweigger for internal monads still adhering to the parent trunk. In other infusoria, as the *Rotifer vulgaris*, the alimentary canal is in the form of a slender tube, and extending nearly the whole length of the body, and terminating at its anal extremity in a dilatation of cloaca for the reception of the ova and the male seminal fluid, previous to its termination at the surface of the animal. Others of larger dimensions, as the *Eosphora najas* and *Hydatina senta*, and in general all the natural group of the *Rotatoria*, possess a single cavity of considerable size and oval form, situate in the anterior part of the body; the *Zygotrochis nudis* would seem to form an exception to the general rule of this division; for this animal, when filled with coloring matter presents a slender, spirally convoluted intestine in the centre of the body. In this animal also, the posterior cloacal dilatation is enlarged into a considerable cavity, which can retain the coloring matter for some time previous to its being discharged by the anus.

The number of stomachs varies no less than their form. The whole tribe of the *Rotatoria*, as already observed, possess but a single cavity. In the *Monas termo*, four can be reckoned.

The number of sacs, which are so many distinct digestive cavities, although connected together by a common tube, varies from 1 and 200 down to 36 in many *Vorticellæ*. The largest number is in the *Paramæcium chrysalis*, Muller, where it amounts to 120, and yet there is ample space for still more.

The *anus* is easily distinguished from the mouth, when the animal is filled with colouring matter, by its discharge from this orifice, in large irregular coherent masses, very different in appearance from the minute state of division in which it enters by the mouth. Its position varies exceedingly; in the greater number, such as the *Hydatina senta*, *Rotifer vulgaris*, and *Eosphora najas*, it opens towards the posterior extremity of the animal; in the first of these it is on the back. In the *Kolpoda cucullis* it opens into the concave surface of the animal, close to the mouth, from which it is only separated by a tongue-shaped eminence. In some of the spirally pedunculated vorticella, its disposition is very singular, opening along with the mouth into a common fissure, which is not situate in the centre of the circular ranges of ciliæ which surround the anterior extremity of the body, but towards the margin, between two of these concentric circles.

The *mouth* merits the notice of the systematologist, from the very precise characters which he can draw from thence for his subordinate divisions. This organ reaches its greatest complication in the *Hydatina senta*, where it consists of an orifice opening in the centre of a globular head, and provided with a pair of serrated mandibules, each resembling somewhat the single mandibules of some of the mollusca, such as the common *Helix pomatia*, or those of the echini. When the animal is in the act of taking its food, these mandibules are in perpetual motion, opening and shutting with great rapidity, to absorb the coloring particles brought within their reach by the currents excited by the motions of the ciliæ. This very singular organization is certainly one of the most curious phenomena visible in their whole structure, and is perhaps one of the most important, as showing so close an approximation to animals far removed from them in the zoological series. Each mandibule in the species which I examined, possessed five distinct teeth, but the number varies from two, three, as far as six. Dr. Ehrenberg has since succeeded in demonstrating their real nature, by the use of very fine foliæ of mica, (the whole animal is not more than one eighth of a line in length,) and has come to the conclusion that they are separate, simple, hard bodies, enveloped with a fleshy covering, which are ingrained into one another like the fingers of the hands when joined.

The mouth of the other infusoria is a simple unarmed opening surrounded more or less closely with a greater or less number of ciliæ.—Its position generally determines their anterior extremity. In the genus *Paramæcium*, it is in the middle of the length of the animal.—The *Kolpoda cucullus* possesses a sort of lip surrounding its margin.

The ciliæ play a very important part in the economy of this class of animals. They may be considered as the principal organs of taste, of touch, and of propulsion. When the animal is at rest, they are often quite imperceptible, but on the addition of a small portion of coloring liquid to the drop of water, they become very apparent, being in a state of great activity, seeming to be the principal agents by which they excite those currents which afford so beautiful a spectacle under the field of the microscope.* In the *Monas pulvisculas*, and other larger monads, their number amount to 10 or 20, and we may from this conclude that they exist even in the smallest monad. They sometimes surround the mouth in a single row, (*Vorticella convallaria, Rotifer vulgaris,*) sometimes in a double row, (*Vorticella citrina;*) occasionally they extend in regular lines, or are irregularly dispersed over the whole surface of the body. The former disposition occurs in the *Leucophrys pyriformis* and *patula*, the latter in the *Actinophrys sol.* They occupy, in other cases, only one side of the body, (*Kolpoda cucullus.*)

An *æsophagus* can only properly be said to belong to those which, like the *Eosphora najas*, and *Hydatina senta*, possess a notable contraction between the mouth and the stomach. This is especially distinct in the latter, where I have distinctly traced the passage of individual colored globules along this narrow canal from the mouth to the intestine.

Perhaps this is the most appropriate place to notice an organ of a very obscure nature, which Dr. Ehrenberg dignifies with the name of a pancreas. It is in the form of two kidney-shaped, grayish-white, glandular-looking transparent bodies, which are placed on each side of the upper extremity of the intestine, firmly connected to, and closely embracing it. Dr. Ehrenberg regards them as bearing a greater analogy to the pancreas than to the liver of the higher animals, from their color, form, and connexions. They must, however, be left to further inquiries.

*One of the most favorable moments for seeing these ciliæ to advantage, particularly in those species in which they invest the whole surface of the body, is when the drop of fluid under the microscope is nearly dry, when they may be seen elongated to their utmost, in a state of great activity; or if the animal be nearly expiring, in a state of rigid erection.

2. *Muscular System.*—A fibrous muscular tissue being the proper agent of all voluntary contraction in the animal kingdom; we might, *a priori*, expect its existence in the class of infusoria, which are so remarkable for the rapidity and energy of their movements of propulsion and translation. In the former they can only be compared with fishes, and in the latter with insects. Contractility of tissue can never explain those active voluntary efforts by which they avoid obstacles when swimming in myriads in a single drop, convey the nutriment towards the mouth, and perform the acts of deglutition. Previous, however, to Dr. Ehrenberg nothing like the muscular fibre had ever been attempted to be shown in these animals.

As yet, from their extreme tenuity, no distinct fibres have been detected in the second and more minute division styled by Cuvier Homogeneous Infusoria, and the new system of Dr. Ehrenberg *Polygastrica;* although from their extremely vigorous contractions, as well as from their presence in the division of the Rotatoria, we are entitled to infer their existence. In this last, distinct fibres are perceptible in the *Eosphora najas*, *Rotifer vulgaris*, *Philodina erythrophthalma* and *Hydatina senta*.

We shall select the muscular system of the latter, the *Hydatina senta*, as a specimen from its greater distinctness and complexity.—The perfectly transparent gelatinous body of this animal, when seen through a microscope with a power of 380, appears to be traversed longitudinally by several narrow bands of fibres, perfectly transparent, and of a grayish-white color. When the animal throws itself into its violent lateral contortions, the fibrous bands are observed to shorten, become broader and thicker, (from their slightly diminished transparency,) on the side towards which the contractions are made; and on the convex to become so extremely elongated and attenuated as to be almost, in some cases quite imperceptible. The real muscular nature of these organs, and that they are the real agents in effecting the motions of the animal, is thus placed beyond all doubt. These muscles never lose their apparent state of tension, which they would undoubtedly do on the contractions of the animal, if their nature was of another description; and when the two extremities of the body are equally approximated to each other, none of the bands become invisible, but all increase to nearly twice their former breadth, with a corresponding diminution of their transparency.

The envelope of the body of the hydatina consists of a double transparent membrane, the two layers of which are in contact with, and scarcely distinguishable from, each other, when the animal is in a state of repose. But, upon the contractions of two or more of the muscles, the internal membrane into which they are inserted becomes separated to a greater or less distance from the external. During the whole of these phenomena, the stomach, ovaries, and the whole of the viscera, are perfectly visible through the transparent muscles.

These principle muscles are four pairs, which take their origin from the opposite ends of the animals, and proceed in a radiated manner to be inserted by broad striated bands near the middle of the body, (between the fourth and fifth pair of twigs given off from what Dr. Ehrenberg calls the great dorsal vessel.) The four upper or anterior muscles rise by narrow insertions from the junction of the head with the body at the root of the rotatory organs; the four posterior or inferior, from the point of insertion of the bifid tail into the body. The extent of insertion of these muscles is much greater in the *Eosphora*, *Philodina* and *Rotifer*, than in the *Hydatina*; in them it reaches at least from the second to the sixth of the above-mentioned transverse twigs.

These great longitudinal muscles are distinct to the most unpracticed eye, but Dr. Ehrenberg views as of a muscular character, 1. The seventeen sections of the rotatory organ in the Hydatina, which must be the principal agents in directing the motions of the cilæ; 2. A contraction or sphincter near the extremity of the cloaca; 3. A striated organ behind the cloaca, which he considers, from its situation, as an accelerator of the seminal fluid, a *musculus ejaculatorius.* In none of these, however, except in the last, can the existence of a fibrous tissue be considered as beyond a doubt; though, from their situation, it is more than probable that this is their true nature. All of these parts seem to be attached to the inner layer of the external double membrane, and to be unconnected with the subjacent viscera. It is not improbable that the tail may possess some proper muscles, as its motions are not performed laterally in common with the trunk, but by an alternate retraction and elongation.

3. *Generative System.*—The partisans of the *generatio spontanea* vel *primitiva,* who so long stood their ground in the class of Entozoa, after being forced to relinquish this position, by the discovery of the ova of these parisitic animals, took refuge in the darkness and obscurity of the miscroscopic infusoria, where they were almost secure of an undisturbed possession, while there was nothing known concerning them except a homogeneous mass of transparent jelley, endowed with a few active motions; and where their negative arguments could only be attacked by analogical reasonings.

The candid and impartial mind of Muller himself, too rigid an observer to be seduced by the allurements of theory, considered the infusory animals as furnishing an incontrovertible argument of the existence of certain living forms, which are neither of oviparous nor gemmiparous organs, but derive their existence immediately from a certain indestructible living generative energy inherent to all matter—for this very plain reason, that he had never witnessed the secrets of their origin. Such a conclusion, though perhaps too hasty, is allowable in such an observer. When, however, we see other men of distinguished talent, such as Treviranus and Oken, taking up the question, where, if it were possible, they ought to have ended, and assume for once the existence of a mysterious power inherent in organic matter of generating infusory and other molecular animalcules, which form by their aggregation all organic living forms, and into which the latter are, at the cessation of their proper vitality, again resolved; we cannot help referring them to the well known maxim of Bacon, that "Homo naturæ minister et interpres tantum facit et intelligit quantum de natura ordine re vel mente observaverit: nec amplius scit, aut potest."

The observations of Dr. Ehrenberg have not only given an additional extension to the great principle of Harvey, *omne vivum ex ovo;* but have, by a connected train of ocular demonstration, proved the existence in this class of the whole three species of generation, the viviparous, the oviparous, and gemmiparous, and even of the simultaneous exercise of two of these in the same individual, at different epochs of its existence. Waiving at present the corroboration which this might give to the view of infusory animals forming a parallel series to their more apparent prototypes, let us proceed to state shortly a few examples of each of these varieties.

In the interior of the *Rotifer vulgaris* we often see young animals of a diminutive size, (that of the parent varying from one-fourth to one-eighth of a line,) perfectly formed, and near the period of exclusion, which already possess the two red points, (eyes,) near the anterior extremity, and a distinct mouth and head. They assume various postures in the interior of the parent trunk, being at times coiled up in a spiral form, or extended to their whole length. These same fœtus, if we may so call them, Dr. Ehrenberg has seen excluded in a living state from the parent. All the individuals of the *Hydatina* are hermaphrodite, possessing the completely formed male and female organs. The female consists of an ovarium, which, when in the unimpregnated state, is an oval perfectly transparent bilobed bladder-like body, closely embracing the lower part of the intestinal tube. When in an impregnated state, it increases very much in size, being augmented by the addition of two or more oval appendages, so that the whole mass fills the greater part of the posterior half of the body of the animal. When quite ready to burst it assumes a greenish-gray color. These rounded bodies communicate by a canal, scarcely perceptible in the unimpregnated state, broad and distinct when nearly ripe, with the cloacal dilatation formerly noticed as existing near the anal orifice of the intestine. That the ova are not internal germs, an opinion entertained by many older observers, such as Lamarck and others, is proved not only from the above-mentioned development and connexions of their containing vesicles, but also by the distinct existence of the three substances which in the ova of the Entozoa, M. Rudolphi considers as the chorion, allontois and amnion. In the centre of many ova there can be recognised a darker point, which is either the embryo, or cicatrix in which the latter is developed.

The adult Hydatina possesses, besides, two organs which Dr. Ehrenberg considers as the male organs of generation, but the real nature of which is a little more doubtful than that of the preceding. They resemble very much in form the milt of fish, consisting of two elongated bodies, extending nearly the whole length of the animal, exterior to the ovaria, broader towards the head, diminishing towards the tail. They terminate, (a strong corroboration of this view of their true nature,) in a number of spirally convoluted tubes, which finally open by two separate canals immediately behind the oviduct. These spiral convolutions are enveloped by an organ of a very singular nature, the functions of which are very obscure: it is oval, transparent, remarkable for its irritability and sudden changes of form; at one time swelling out into a vesicular form, at another contracting into a small glandular looking organ. Dr. Ehrenberg at one time considered it to bear some analogy to a uterus; but it is more probably connected with some office in applying the seminal fluid to the ova previous to the exclusion. This organ is wanting in the *Rotifer* and *Philodina*, where the male apparatus otherwise resembles very closely that of the Hydatina.

In the *Kolpoda cuculus*, the parent animal excludes the ova in the form of extremely minute globules, bearing much similarity to some of the species of the genius *Monas*, connected by a number of fillaments interwoven together in a reticular form. In an animal 1-24th of a line in diameter, that of the ova was 1-1000th of a line. The young Kolpoda were 1-144th of a line before they were distinctly seen to excite currents, and swallow the colored particles. In the genius *Vorticella* there seems to be a combination of the oviparous and gemmiparous generations. The single species *Convallaria*, has from their entire ignorance of its mode of developement, been subdivided into no less than six distinct genera, by different observers, according to the variety in its form at different stages of its existence. It first appears in the form of minute points, not more than 1-1000th of a line in diameter, scattered upon the peduncles of a group of adult Vorticellæ. After a time these minute points enlarge in size, and send out delicate pedencular prolongations to the larger adult roots, in which state Schrank styled them *Vorticellæ monedicœ.* When still more advanced these peduncles become coiled up in a spiral form. And when they may be considered as having reached their complete organic developement, though still much inferior in size to what they afterwards attain, the usual currents may be observed in the colored solutions in which they are immersed. The same species propagates itself by germs, on the separation of a part of its body from the parent trunk.—This is performed in three different ways, each of which has been dignified with the title of a distinct form.

The first is the longitudinal division in which the animal divides itself into two nearly equal halves. A fissure first appears traversing the whole length of the body; this becomes deeper anteriorly, where two horns are now visible, each provided with a distinct set of ciliæ, and a mouth, recognisible by the two currents of coloring particles directed to the apices of the two horns. The fissure becomes deeper and deeper, till they form two distinct, perfectly formed animals, attached to the apex of a single peduncle; one of these is soon detached from the latter, when it agrees in form exactly with Lamarck's genius *Urceolaria.* When the same animal moves with the hinder part forward, it forms Schrank's genius *Ecclissa;* when the conical basis by which it was attached to the peduncle, has not quite disappeared, it forms Bory de St. Vincent's genius *Rinella;* and when a little broader and more bell-shaped, with apparently only two ciliæ, it is the genius *Kerobalanus* of the same author; when fully provided with ciliæ, without any remaining vestige of the conical appendage, it is the genius *Craterina.* This Vorticella also passes through the same phrases of a *transverse* division into two equal independent animals. The third method is the true gemmiform division, as in the Hydræ and Planariæ, in which a small bud is given off from the posterior surface of the animal, which is provided with ciliæ, and when saparated from the parent trunk, is still of a very diminutive size.

Such are a few of the observations on the generation of these animals, from which it will be seen that they are but in their commencement, and that much remains to the patience and labor of future observers.

4. *Vascular System.*—The existence of a digestive, a muscular and, a generative system of much complexity, and very far from what we might consider as their simplest expression, may now be viewed as an ascertained fact with regard to infusory animals. The existence of the two systems which remain for our attention, viz: the vascular and nervous, is as yet somewhat problematical. The organs on which Dr. Ehrenberg confers these appellations, are very apparent, but much doubt exists with regard to their real functions.

What has been denominated a vascular system is distinctly visible only in the *Hydatina senta.* Traces of a similar arrangement are now and then perceptible in the *Eosphora*, in particular positions of the animal, but they quite disappear when the integuments are in a state of strong tension. In the former, a series of transverse lines of a white color, and inferior transparency to the rest of its body, succeed one another at regular intervals, from the head towards the tail. These transverse striæ might at first be taken for muscles, but they differ from these entirely in their aspect and connexions. They are nine in number, exactly parallel to, and nearly at equal distances from, each other. At first sight they seem to be complete rings encircling the whole body; but, upon a closer inspection, they are observed to diminish in breadth, and finally vanish on approaching the inferior or abdominal surface of the animal. On the contrary, they augment in diameter towards the back, where they all terminate at right angles, in a line, of an exactly similar appearance to themselves, running in a longitudinal direction from the head to the tail. This longitudinal line or vessel is nearly twice the caliber of any of its tributary traverse twigs.

It will be observed that, the disposition of this main dorsal trunk, with its collateral branches, is almost exactly that of the vascular system of the Ascidia, so beautifully demonstrated by M. Savigny, which is a strong argument for their being of the same character, No motion of an internal fluid is discernable in their interior, nor has any pulsation, analogous to a heart, been ever observed. Both these phenomena, which would decide the question as to their true nature, Corti asserted that he had observed in the *Rotatoria* and *Brachionus*, but he was deceived by the tremulous motion of the canal, leading from the mouth to the œsophagus. The same was the case with Gruithuysen, who mistook the motion of the intestine in the *Paramœcium aurelia* for that of a sap-like fluid. It is worthy of note, that these white striæ are attached to the internal, not the external tunic of the integuments.

5. *Nervous System.**—This name is given to a series of six or seven round glandular-looking, grayish bodies, which envelope the upper or dorsal part of the œsophagus of the *Hydatina.* They are closely connected together, and are distinguished from all the other viscera of the body by their darker tint. The uppermost of these bodies, (ganglia,) or that scituate in the mesial plane, is much larger than the rest, and gives off, from its apex, a slender branch which proceeds upwards towards the integuments at the back of the neck a little before the second pair of vascular twigs, where it forms a slight enlargement, (ganglion;) it does not stop here, but returns back and unites again, not with the large ganglion from which it was originally given off, but in one of the adjacent smaller ones. A complete circle is thus formed, bearing some resemblance to the nervous circle, which encircles the œsophagus of the mollusca, except that in this case the whole circle is situate on the dorsal or upperside of that canal. From the point of contact of this nervous circle with the dorsal vessel, it gives off two very slender twigs forward to the anterior part of the head, where, in other forms of the Rotatoria, such as the *Rotifer vulgaris*, the two red points, (eyes,) are situate. In some, such as the *Eosphora najas*, a single large red point is situate on the back of the neck, in the exact position of the ganglion at the apex of the circle.† The above-mentioned large mesial œsophageal ganglion, (brain,) sends off posterioly another branch of much larger size, backwards along the abdominal surface of the animal, which closely adheres to the internal layer of its double envelope.

That these different fillaments and ganglia, to which we have given the name of nerves, are not muscles, is evident from their form, their mode of insertion into the integuments, and because in the contractions of the animal they are not shortened, but assume a serpentine form, being apparently quite passive. They are not vessels, because no pulsation nor motion of a contained fluid has been hitherto perceived through their transparent tissues. If they are not organs of an entirely unknown nature, the whole analogy of their form and position, compared with that of the nervous system in other invertebrate animals, favors the idea of this being their true nature.

We may here consider as appendages to the nervous system, those colored points situate in the anterior part of the head of these animals, and most usually on the dorsal surface, which have been considered as

*According to all our ideas of known physiological laws, the existence of active voluntary motion presupposes the necessity of an animating nervous system. Hitherto, however, no attempt had ever been made to prove its existence. But here again in these animals, excluded by their delicacy and minuteness from the ordinary means of anatomical investigation, the transparency of their tissues, as it has enabled us to discover the existence of a muscular, has no less assisted us in the more than probable discovery of its necessary appendage, a nervous tissue.

†The best view of the disposition and appearances of the œsophagael ganglia is got from the dorsal side of the animal, in a line with the great dorsal vessel. The nervous collar given off from the brain, is however, best seen on a lateral view.

eyes. As already noticed, the first discovery of these organs was made in 1816, by Nitsch, who saw in the *Cercaria viridis*, (now referred by Dr. Ehrenberg to the genius *Euglena*,) three black scaliform points. In the *Rotifer vulgaris*, their pigment is of a red color, and they are three in number, two small ones at its anterior extremity, and a single larger one at the nucha in the situation of the apex of the above-mentioned nervous circle in the *Hydatina*; and it is very probable that the two filaments, which in the latter animal are sent forwards from this ganglion, or even the ganglion itself, subserve the purposes of vision. The number, disposition, and color of these points is the same in the *Eosphora najas*, where the mesial eye is still larger and more distinct. In the *Philodina erythrophthalma* their color is the same, but they are only two in number, (the most common disposition in this class,) much smaller, and situate more posteriorly. In the *Lapadella ovalis* one only is visible of considerable size in the mesial situation of the large one of the Eosphora.—*Amer. Jour.*

2. *Physiological Investigations arising from the Mechanical effects of Atmospherical Pressure on the Animal Frame.*—The weight of the atmosphere, though not constantly the same, varying from 1-12th to 1-15th of its whole weight*, on an average amounts to fourteen and a half pounds on each square inch of surface; and to this pressure the human body in common with all other objects is subjected. The whole surface then of a middle-sized person will have to support from fifteen to twenty tons of pressure, all acting inwards and tending to compress the body into a less compass. Now the question arises, how is it that the animal frame is utterly insensible of the whole, or of any part of this enormous pressure upon it. Mr. Dalton, in a late volume, (Vol. X.) of the Manchester Memoirs, has attempted a solution of this question, and we shall lay before our readers the views he has presented.

It is pretty well known that the *specific gravity* of living men in general, is less than that of water.† Mr. Robertson, formerly librarian to the Royal Society, found that if ten men taken promiscuously, one of them was a little heavier than water, and two nearly the same weight as water, two others were only about .8 the weight of water—and the other five were of intermediate specific gravities. The average of the ten was—height, 5 feet 6 3-4 inches—weight 146 lbs.—specific gravity, 1.891—bulk, 2.618 cubic feet. From this Mr. Dalton thinks that it may be safely inferred, that the body of a full-grown living man, when plunged over head in water, will be found to average nearly .9 of the weight of an equal bulk of water.

"It is remarkable," observes Mr. Dalton, "that all the component parts of the animal frame, as least of the human subject, are severally specifically heavier than the whole body, with the exception of air.—Bone, muscular flesh, blood, membrane, &c., are all heavier than wa-

* These variations are gradual, so that it requires some days or weeks before the weight passes from one extreme to the other.

†Phil. Trans. Vol. 50.—Hutton's Dict. Sp. Gr.

ter: animal fat is, perhaps the lightest of the components, but even this is heavier specifically than the whole man upon the average.—Bone from the leg of a calf I found to be 1.24 specific gravity. The lean of beef, (raw,) I found 1.045 specific gravity. Blood is from 1.03 to 1.05 specific gravity according to circumstances:—on the whole, the solid and the liquid parts of the body examined after life is extinct, would appear on an average to be somewhere about 5 per cent heavier than water.

"That part of the volume of man which is exclusively occupied by air, and which may therefore be considered as adding nothing material to the weight of the body, consists of the air-tubes and air-cells of the lungs, the trachea or wind-pipe, the mouth and other appendages. It is not easy to ascertain the medium volume of air in the lungs of any individual. Messrs. Allen and Pepys found the air remaining in the lungs of a man after death somewhat exceeded 100 cubic inches. I found formerly that after a full inspiration I could blow out 200 cubic inches of air from my lungs, but was then quite exhausted. My ordinary inspirations and expirations amounted each to about 30 cubic inches.*

"Judging from the above facts and considerations I should be disposed to conclude that the medium volume of air in the lungs of a middle-sized person would not be less, but rather more than 100 cubic inches. Besides the lungs there are no other receptacles for air, I believe, in the body, except the stomach and bowels, which are occasionally more or less inflated with portions of air either from the atmosphere or from other sources. If we allow 150 cubic inches for the volume of air, contained in the whole man when entirely immersed in water, it will be as fair an estimate perhaps as can be made. But it may be imagined by some that the whole surface of the body is pervious to air; that the skin, the flesh, the blood, and even the bones, may be imbued with air, somewhat in the same manner that water is, and yet have no cavities or cells in which the air is collected into a visible volume. Whether such an idea has ever been entertained or discussed I am not aware; but I presume no one has succeeded in determining either the nature or the quantity of the air so enveloped in the system. We shall now examine how far such a notion is countenanced by the preceding statements of facts.

"According to the preceding table of Robertson the average bulk of the ten men was 2.618 cubic feet—4500 cubic inches nearly; but of this volume 150 inches according to the above estimate were air, and the remainder 4350 inches were solid and liquid parts of the body. Now the average specific gravity of those parts of the body has been estimated above at 1.05 when examined as dead matter; this would make their weight equal to 4567 cubic inches of water; whereas it was found by actual weighing to be 146 lbs. as per table—4044 cubic inches; hence the observed weight was less than the calculated weight, a portion equal to the weight of 523 cubic inches of water, or more than 1-9th of the whole weight of the body."

*Memoirs Vol. II. (new series, p. 26)

This discrepance demands investigation. Mr. Dalton thinks that it is not likely that Robertson's table of the specific gravities of men give too low an estimate; be this as it may, it certainly is not true as stated by him, that the human subject generally floats in water *until the lungs become filled with that element.* Dr. Cullen in many dissections found no water in the lungs; and Dr. Goodwyn's experiments which are conclusive on this subject, show that only a very small quantity of water enters the lungs of drowned animals.* The specific gravities of the component parts of the body appear not to be overrated—and it will hardly be contended that the lungs of a middle-sized man will hold a medium state of inflation six times the volume of air Mr. D. has assigned.

Upon the whole, Mr. Dalton "is inclined to believe the true explanation of the difficulty will be found in this, that the whole substance of the body is pervious to air, and that a considerable portion of it constantly exists in the body during life, subject to increase and diminution according to the pressure of the atmosphere, in the same manner as it exists in water: and further, that when life is extinct, this air in some degree escapes and renders the parts specifically heavier than when the vital functions were in a state of activity.

"The facts that water absorbs air of all kinds, that the quantity of air absorbed is proportional to the pressure and density of the gas, whether it be alone or mixed with other gases, and that certain laws of equilibrium take place, by which water acquires that state in which it is disposed neither to give out nor take in any more gas, have been abundantly proved by Dr. Henry and myself. M. Saussure has shown the like for other liquids, and for a great number of solid bodies. It may be seen, too in my Chemistry, vol. 1st, page 236, that a bladder, which is generally considered as an animal membrane least pervious to air, may be filled with one gas, and being sometime exposed to the atmosphere, it will be found to continue full blown as at first, but the contents will be chiefly atmospheric air. Messrs. Allen and Pepys in their ingenious and excellent essay on respiration, have proved that when a Guinea pig, or a pigeon is confined, for an hour, more or less, in a mixture of hydrogen and oxygen gases in proportion as 78 to 22, a large portion of azotic gas is found in the residue and an equal portion of hydrogen disappears. They ascribe this change to effects of respiration; but it appears to me more probably due to the principle we are advocating; namely, to the egress of azote from the whole body, and the ingress of hydrogen in lieu of it, in consequence of withdrawing the external pressure of the former and substituting that of the latter.

"When the palm of the hand is placed over the top of the receiver of an air-pump, and the air is exhausted, the pressure of the air on the outside is scarcely felt, but the inside is swollen and feels as if it was drawn or sucked into the receiver. Thus the sensation is on the inside and not without; the reason is, there is no change of pressue on the outside; but there is within, and the consequence is a tendency of

*See article Asphyxia in the forth-coming Medical Cyclopedia.

the air in the hand to escape into the receiver, which occasions the pain and swelling. It is thus also that the issuing of blood in the surgical operation of cupping is effected.

"Though it does not seem of much consequence what the pressure of the air may be on the animal frame, within certain limits, yet sudden changes must always be accompanied with uneasy sensation. Climbing mountains, or ascending in a balloon, removes a part of the atmospheric pressure from the body; this causes the air in the body to tend outwards, and sometimes occasions bleedings. To supply oxygen to the lungs a greater volume of air must be breathed, and this seems to produce an acceleration of the pulse. On the other hand, by descending thirty or forty feet deep into the water in a diving bell, the pressure of the air upon the body is increased inwards; pains in the ears are felt from the difficulty of suddenly restoring a disturbed equilibrium; but if the descent is slow and interrupted, time is given for the air to enter the pores, and the pain is less sensible. To what limit warm-blooded animals could bear the rarefaction of air so as to subsist, has not that I am aware of, been determined with much precision.—Ascents in balloons have been made till the atmospheric pressure was reduced more than one-half. Formerly I found that a mouse could subsist in air of one-fourth atmospheric density, and seemed not to have suffered much; but upon reducing the density below one-fourth, the animal was convulsed and expired immediately, notwithstanding the air was instantly admitted.

"If the view we have expanded in this essay, in regard to the action of ariel pressure on the animal frame, be correct, it may be inferred, that the pressure admits of great latitude; perhaps an animal could subsist under the pressure of one-half an atmosphere, or of three or four, or more atmospheres. The uneasiness and danger would be found in the quick transition. If time is allowed for the air to enter the body, and to escape from it, the transition is gradual, and the sensation arising from it imperceptible. The animal economy would be adapted to it, like as in the transition from a cold to a warm climate. It may hereafter be found, what length of time is sufficient to adjust the equilibrium, and whether this subject is any way connected with certain diseased states of the body. As far as regards the absolute pressure on the body, and our insensibility of it generally, this question will be met by the argument, that the air within the body, by its elasticity, sustains a corresponding pressure from without; but this only accounts for our alleviation from a small fractional part of the whole exterior pressure. The greater part must still be supported by the body; and we must have recourse to the great incompressibility of matter to account for our insensibility of pressure. Canton found that water, pressed by one atmosphere more than ordinary, only exhibited a reduction of 1-21740th part of the whole; if the same rate, applied to the compression of the human body, the reduction or compression of the size of a man, 4500 cubic inches, would only be 1-5th of a cubic inch, for the weight of an additional atmosphere. Now as the body consists of solids and liquids of almost incompressible matter, and there is only a small part of the volume consisting of elastic fluid that is compressible, no material change of volume can take place, but on the sudden transition from one atmospheric pressure to another, and unless a change of volume take place, we cannot feel any pressure, either inward or outward. The phenomena of the water-hammer show, that the particles of water are hard, as they strike each other like flint and steel; and it is exceedingly probable that other bodies, solids as well as liquids, are constituted in like manner. A general pressure on the system then, only increases in a small degree the attraction of the ultimate particles, and it is met by a corresponding increase of repulsion from the atmosphere of heat; so that the system remains, as nearly as possible the same, and unaffected by such pressure."

3. *Quantity of Food taken by a Person in Health compared with the Quantity of the different Secretion during the same period.*—The Fifth Volume of the *Memoirs of the Literary and Philosophical Society of Manchester,* contains an account of a very interesting series of experiments on the quantity of food, taken by a person in health, compared with the quantity of the different secretions during the same period; with chemical remarks on the several articles; by JOHN DALTON, F. R. S. The first series of experiments was made upon himself, in the month of March, for fourteen days successively. The aggregate of the articles of food consumed in the fourteen days was as follows: of bread, 163 oz. avoir. oat cake, 79 oz.; oat meal, 12 oz.; butcher's meat, 54 1-2 oz.: potatoes, 130 oz.; pastry, 55 oz.; cheese, 32 oz.; total of solid food, 525 1-2 oz. avoir.; averaging 38 oz. daily. Of milk, 435 1-2 oz.; beer, 230 oz.; tea, 76 oz.; total, 741 1-2 oz. avoir.; average 53 oz. of fluid daily. Thus it appears that the daily consumption was 91 oz.; or nearly 6 lbs. During the same fourteen days, the total quantity of urine passed was 680 oz.; and the fæces, 68 oz.; the daily average is consequently of urine, 48 1-2 oz.; of fæces, 5 oz.; making 53 1-2 oz. The quantity of food taken daily having been 91 oz., there remains 37 1-2 oz. to be accounted for, which must have been spent by the insensible perspiration from the skin, and that from the lungs conjointly, on the supposition that the weight of the body remained stationary.

To try the effect of different seasons, Mr. Dalton resumed these investigations in the month of June the same year, and continued them for one week successively. The results were what might have been anticipated; a less consumption of solids, and a greater of fluids; a diminution of the evacuations, and an increase in the insensible perspiration. The following were the results: solids consumed in seven days, 236 oz.; average, 34 oz. per day; fluids, 391 oz.; average, 56 oz. per day. Total per day, 90 oz.; or 4 oz. less per day in solids; 3 oz. more in fluids than in the former trial. The daily averages in the evacuations were, urine, 42 oz.; fæces 4 1-3 oz.; leaving a balance of nearly 44 oz., for the daily loss by perspiration, being an excess of about 6 oz. above that in the former season, or one-sixth more, owing no doubt to the higher temperature.

Another trial of a week's duration was made in September, with results nearly similar to the last. The daily consumption of food was, 93 oz.; and the perspiration nearly one-half that quantity.

Mr. Dalton next varied the process, with the view to obtain the quantity of perspiration and the circumstances attending it more directly. He procured a weighing beam, by which he could weigh his body, so that the beam would turn with one ounce. Dividing the day into periods of four hours in the forenoon, four or five hours in the afternoon, and nine hours in the night, or from ten o'clock at night to seven in the morning, he endeavored to find the perspiration corresponding to these periods respectively. He weighed himself directly after breakfast, and again before dinner, observing neither to take or part with any thing during the interim, besides what was lost by insensible perspiration; the difference in the weights, in this case, was the loss by perspiration. The same procedure was adopted in the afternoon and in the night. This train of experiments were continued for three weeks in November. The mean hourly losses by perspiration were, morning, 1.8 oz. avoir.; afternoon, 1.67 oz.; night, 1.5.—During twelve days of this period, Mr. Dalton kept an account of urine, corresponding in time with perspiration. The ratio was as forty-six to thirty-three, which is somewhat a greater disproportion than that observed in March; probably owing to the lower temperature of the weather in November.

These experiments were made forty years ago, but animal and vegetable chemistry being at that time in their infancy, no deduction of any interest could be made. The progress which this branch of physiology has since made, enables us now, however, to approximate very closely, to the quantities of the several chemical elements to be found in the great variety of products of the two kingdoms.

Bread and farinaceous vegetables appear to constitute the greatest part of ordinary food. Mr. D. has found that 5 lbs. of flour make 7 lbs. of bread. Now from this analysis of flour, Mr. D. estimates the carbon in bread at 30 per cent. Twelve ounces of bread, (the daily average in his first set of experiments,) must then contain 3.6 ounces of carbon. Seven ounces of oat-cake and oat-meal he estimates to contain 1.8 ounces of carbon; four ounces of pastry to contain one ounce of carbon; nine ounces of potatoes to contain nearly one ounce of carbon; four ounces of butcher's meat, and two ounces of cheese, together about three ounces of carbon;* thirty one ounces of milk, eleven-twelfths of an ounce. Twenty-two ounces of tea and beer would contain only a small fraction of an ounce of carbon, not easily estimated, and of little account by reason of its smallness. The amount of the element carbon then in some state of combination taken into the stomach, by different kinds of food, was 11 1-2 ounces.

According to Berzilius, the urine of healthy persons differ materially according to circumstances, but upon an average it may be reckoned to consist of 93 or 94 per cent of water; the rest consists of many substances; the carbon contained in which cannot be estimated at more than one or one-fourth per cent. from the analysis hitherto made. This will give .5 or .6 of an ounce of carbon in 48 1-2 ounces of urine per day. According to the same chemist, fæces may be es-

*According to Gay-Lussac's experiments.

timated as containing 75 per cent. of water, and the remainder does not seem to contain more than ten parts of carbon. This would give half an ounce of carbon in five ounces of fæces. Hence we may infer that one ounce, a little more or less of carbon, is carried off from the body daily through these two channels. The remainder 10 1-2 ounces must therefore be spent in the insensible perspiration.

The quantity of insensible perspiration from the skin cannot be easily determined by direct experiment; but that from the lungs may be approximated from known facts.

Mr. Dalton has shown* that he produced by breathing in twenty-four hours, 2.8 lbs. troy of carbonic acid gas—.78 lbs. troy, of carbon=.642 lbs. avoirdupois,=10 1-2 ounces nearly. He has also determined† that the quantity of aqueous vapour which he exhales from his lungs does not exceed 1.55 lbs. troy,=1.275 lbs. avoirdupois,=20 1-2 ounces avoirdupois. If to this we add 10 1-4 ounces of carbon, we have 30 3-4 ounces for the carbon and water expended from the lungs in one day, and this taken from 37 1-2 leaves 6 3-4 ounces per day, for the insensible perspiration from the skin, which if the above estimate be allowed, must consist of 6 1-2 ounces of water, and 1-4 of an ounce carbon. According to this, the matter perspired from the lungs is five times as much as that from the whole body.

If instead of carbon, we trace the element azote into and out of the body, it will appear that from the butcher's meat, cheese and milk, about 1 1-2 ounces of azote was taken into the stomach daily, and nearly as much passed off by urine and fæces.

"Upon the whole we may observe," remarks Mr. Dalton, "that of the 6 lbs. of aliment taken in a day, there appears to be nearly 1 lb. of carbon and azote together; the remaining 5 lbs. are chiefly water, which seems necessary as a vehicle to introduce the other two elements into circulation, and also to supply the lungs and other membranes with moisture. Very nearly the whole quantity of food enters into the circulation, for the fæces constitute only 1-18th part, of these a part bile, must have been secreted; one great portion is thrown off by means of the kidneys, namely, about one-half the whole weight taken, but probably more or less according to climate, season, &c.; another great portion is thrown off by means of insensible perspiration; this last may be subdivided into two portions, one of which goes off by the skin, amounting to one-sixth part, and the other five-sixths are discharged from the lungs in carbonic acid, and in water or aqueous vapor."

4. *Experiments on Hemlock and Henbane.*—Professor Geiger, of Heidleberg, whilst recently engaged in making chemical experiments, succeeded in establishing some remarkable illustrations of the active principles of hemlock. Its base is an organic salt, which opens an entirely novel series of these highly interesting organic substances, for it is volatile, and similar to a volatile oil. The peculiar qualities of this substance, both intrinsically, and when brought into combination with acids, its rapidly changeable character, and the brilliant play of colors

*Manchoster Mamoirs, Vol. II. New Series. †Ibid.

which it exhibits whilst undergoing change, render it one of the most interesting productions in organic chemistry. Its poison is of the deadliest description. The smallest quantity, applied inwardly, produces paralysis; and one or two grains are sufficient to kill the largest animal. Another of Professor Geiger's late discoveries is the active principle of henbane, (*atropin*,) its base is likewise an organic salt, but it is tenacious, admits of being reduced to a crystal, forms a crystalline salt with acids, like hemlock, and has a disagreeable smell, though it is not volatile, unless it be subjected to a decomposition.—Its poison is quite as deadly as that of the former, but exhibits dissimilar appearances, and is not so rapid in its effects. Animals, where even a minute dose is administered, become languid, cannot stand upon their legs, are attacked by convulsions, and die within six hours. The effect of this poison in dilating the pupil of the eye, is extremely remarkable. The minutest portion of it, when applied to the eye of a cat, produces a dilatation of the pupil for the next four and twenty hours; and the hundredth part of a grain prolongs the appearance for the next seven or eight days, besides inducing other singular symptoms of poisoning.—*Rep. Pat. Invent. March*, 1833.

5. *Cold Dash in Convulsions of Infants.*—The application of a small stream of ice-cold water to the head, is recommended by Richter in his *Specielle Therapie*, as very successful both in the convulsions and coma of hydrocephalus. This practice is also pursued by Dr. Heim, of Berlin, and repeated so long as the fits of insensibility continues. The neck and shoulders ought to be covered with oiled silk, and the body kept warm. Dr. Graves, of Dublin, recommends a similar practice.—*Dub. Jour.*

6. *On the excision of Hæmorrhoidal Tumors.*—In our last No., p. 513, we gave an extract from a clinical lecture of Baron Dupuytren's on this subject. In a subsequent lecture the Baron offered some interesting remarks on the consequences of the excision of hæmorrhoidal tumors. These remarks are so important that notwithstanding their length we give them in full. We take them from the report in our cotemporary, the *London Medical and Surgical Journal.*

Dr. Marx has inquired of me, observed M. Dupuytren, if we should not *always, and in every case cauterize immediately after the operation*, rather than run the chance of internal hæmorrhage, which presents the serious danger we have already explained. I agree with him, for it results from the recapitulation of a great number of hæmorrhoidal extirpations, which I have performed at the hospital, as well as in the city, that this consecutive internal hæmorrhage came on unexpectedly in two-fifths of the cases of operations which have not been cauterized; never on the contrary, has it taken place in cases where the cautery has been used. The question then to be decided is, are the inconveniences of cauterization preferable to the dangers to which the patient is exposed from hæmorrhage? Now it may be remarked to me, that no comparison can be made between them, that the inflammation and tumefaction which occur after cauterization, the irritation which extends to the rectum and urinary organs, generally yield to the simple treatment which I have before pointed out, and have never been followed by fatal effects; while on the contrary, internal hæmorrhage puts the patient's life into the most imminent danger. Let us suppose a case where some circumstances would not permit assistance to be given in time to the patient attacked by internal hæmorrhage, he will perish, and the operator will feel the greatest regret for not having prevented this accident by using cauterization. Finally, it may be said to me, that since this hæmorrhage occurs in a great many cases, and that it is impossible to know *a priori*, if the patient that is to be operated on may be one of the few who escape this accident, why not admit it as a principle that cauterization should always be used. I acknowledge that these considerations appear to be just, and they will lead us without doubt to modify the treatment which we have used to this day in these cases. A treatment as certain in stopping hæmorrhage, is the introduction of a pig's bladder stuffed with charpie into the anus. Though it succeeded in the first operation of this kind that I performed, says M. Dupuytren, I preceived that it was very annoying to the patient, and that it was almost always expelled involuntarily by spontaneous efforts, which were induced by its presence.—The other consequences of the excision of hæmorrhoidal tumors are much less dangerous and less unpleasant. There frequently appears a considerable tumefaction of the cellular and adipose tissue of the anus; the principal inconvenience attending this tumefaction is the severe irritation of the rectum, in consequence of which the patient, during four or five days after the operation, feels it quite impossible to go to stool: but the aperient and the enema being again administered, strict regimen being observed, this want will be very much moderated, and will remove a constipation of some days, which would be otherwise annoying. This tumefaction may also cause a retention of urine, but we possess most efficacious means of removing this; as for the tumefaction, it yields quickly to the application of leeches, emollient fomentations, baths, &c. The pain attendant on excision is intense, but almost instantaneous, and this annoyance, which is inseparable from the slightest operation, should not be weighed against the pain and danger caused by the disease. After the operation the patient is liable to different affections, which ought to be the special objects of the surgeon's attention, and which it is in his power to prevent.

It must be acknowledged that persons affected with disorganized hæmorrhoids; are reduced to a state of profound anemia, to asthenia, brought on by the abundance and frequency of hæmorrhages or seropurulent discharges. These evacuations, to which the patient has for a long period been subject, cannot be suddenly stopped without causing a reaction in the whole systen; a general state of *artificial* plethora is induced, sanguine congestions take place in the lungs, liver, and brain, and affections of these organs may follow. The patient is often seized with syncope, spasms, giddiness, and falls into a state of alarming insensibility; the arteries pulsate with such violence that one would think that there was aneurismal diathesis, if these anormal pulsations were not changing every instant their seat and form; and it is a remarkable fact, that this plethora coincides with a pale complexion, the skin generally yellow or earthy colored, especially the face, and with great weakness. Repeated bleeding for sometime, and at short intervals, if the patient is young, vigorous and robust; and if the discharge from the anus has been sanguine, the introduction of a seton and of a cautery, if the discharges were of a purulent nature; these two remedies combined, if the case require them, gentle laxatives frequently administered. These are the most approved remedies, and this is the most rational prophylactic treatment we can make use of to prevent a plethora, the existence of which would lead to the most serious danger. When the excision of the external tumor is performed, the cicatrix which remains, either from constriction of the sphincter, or from the tension of the teguments and of the anus, is sufficient in the greater number of cases to oppose efficiently the protrusion of the internal tumor, and we can then dispense with having recourse to the excision of the latter. Beside, the second excision, like that of the external tumor, is generally without injury, and the patient is completely cured of the disease. Excision may sometimes be followed by contraction of the anus. J. L. Petit has reported a case where the contraction was such, that the pipe of a syringe could scarcely be introduced; and this accident can be prevented by introducing into the intestines large wicks, and by renewing them until the cure is perfect. Let us now apply the directions given by M. Dupuytren, his particular practice, and that of his hospital will furnish us with a number of cases. 1st observation.—A shoemaker about thirty years of age, came sometime since for advice for hæmorrhoidal tumors, which weakened him very much; his trade obliged him to sit continually bent; but he attributed his disease to a visit he made in Champagne, where he indulged in numerous excesses in the wine of that province. It was at that time in fact, he first perceived that tumors had formed at the verge of the anus. They were at first very small, very slightly painful, and only protruded when the patient went to stool; they afterwards increased considerably in size. These tumors, as in many other cases, presented two stages, one which may be called inert, during which time the hæmorrhoids did not discharge, or the discharge was a slight serous oozing, without any inflammatory symptoms; the other stage, designated hæmorrhoidal crisis, is perceived by swelling inflammation, severe darting pains, a considerable discharge of blood, afterward of sanguineous serosity. These crisis returned more frequently, their duration increased, the sufferings of the patient became more violent, and his health suffered great injury. When he came to the hospital he was weak, emaciated and yellow; he walked quite bent, and could not straighten himself. This position resulted from a considerable protrusion of the hæmorrhoids, which was at least as large as the fist of a child of seven or eight years old, and was composed of two tumors, one internal, and the other external. The patient was besides affected with obstinate constipation, which is often the consequence of an irritation, which extends itself to the rectum, and of a retention of urine, a complication not less common than the preceding,

M. Dupuytren recommended the administration of enemas, and the use of baths; by these means the retention of the urine was removed, but the fæcal matter remained. The swelling of the hœmorrhoids was much diminished, there was less redness, the patient suffered much less. There is no doubt that leeches, emollient fomentations, baths, enemas, rest, and proper drink, will not cure the actual crisis, but it is evident that such treatment will palliate it, and that accidents will reproduce this affection at an epoch more or less distant, according to the hygenic state of the patient.

We may be asked, perhaps, what inconvenience would result from the use of this palliative treatment at each return of the crisis? It is this treatment adopted by many physicians; it is also preferred by many patients who dread the operation. It sometimes happens that the temporary relief retards the return of the crisis, and renders them more unusual; but more frequently they reappear, and the health of the patiant visibly alters.

This motive, however allowable it may be, should not be put in comparison to the dreadful effects which the continuance of the disease would bring on; the tumours, external as well as internal, often become schirrhous; sometimes the latter, on its development, recedes up the rectum, to a height which we cannot attain, and the disorganization extends itself in the inside of the intestines. If to these disagreeable consequences you add the general state of the patient, who presents a severe affection of the system, you will think with me, says M. Dupuytren, that it is necessary to práctice excision in the actual crisis. But again, do not think that in giving this opinion, I mean that the extirpation of the hæmorrhoids should always be practised; I have pointed out before in what circumstances they should be left to nature, and when they should be removed by cutting instruments. After these preliminary considerations, M. Dupuytren directed that the patient should be brought. He lay on the bed on his knees and elbows, his thighs separated, and with the scissors before mentioned, the professor excised the hæmorrhoidal tumors; after the excision the wound was not cauterized. Cauterization, though certain in its results, has something appalling to the spectators. I have seen you shudder more than once at the sight of the red iron, and the cloud of smoke which rises from the cauterized part; you may judge what an impression such a preparation would produce on the friends and relations of the patient, who are not, like you, accustomed to such scenes.

Meanwhile, fearing that the hæmorrhage would supervene, we recommended the surgeon of the ward to watch the patient with the greatest care, and to apply the cautery if the blood began to flow in the rectum. It was also to avoid this disastrous occurrence, that we made it a rule not to apply the dressings for some hours after the operation, because it is to be feared that the dressings would only hinder the blood from flowing out, and thus cause it to flow back into the superior intestines.

What we apprehended, continued M. Dupuytren, happened the next day, an internal hæmorrhage manifested itself; the pupil of the ward was not mistaken, from the symptoms we had so plainly pointed out; he had recourse to the means that have always succeeded with us.—He gave him an enema, which brought away a great quantity of blood, a second enema brought a considerable clot; he then made the patient strain, first, to expel any blood that might remain, and secondly, to cause relaxation of the sphincter, and exhibit the surface of the divided arteries; then he applied to the bleeding parts two red hot iron instruments. The hæmorrhage did not reappear, and from this period the patient no longer experienced cholic pains, or syncope.

The quantity of blood lost in this operation has been estimated to be three, four and five pounds. It flows into the descending colon, also the transverse and ascending, and as far as the cœcum, but never beyond this. The patient, whose case we are describing, presented a complete assemblage of the symptoms of the disease, and the consequences of the operation. From the effects of cauterization he experienced a retention of urine, when it was necessary to use the catheter; after the evacuation of a great quantity of urine, he feels great pain, which does not cease till the organ returned to its usual state. But the inflammation and swelling caused by cauterization are already diminished, the patient is going on well, and in about fifteen days he will be cured.

Finally, we know that persons affected with hærmorrhoids are subject to obstinate constipation; in this case it lasted for several days; excision as it often happens, had increased it. We have already remarked, that we should not induce stool until the inflammation and swelling have decreased or even disappeared; because before that time, the fæcal matter cannot be expelled without causing violent pain, augmenting the irritation, and tearing the parts. It was not until after this time that enemas and gentle aperients were administered.—The sixth day after the operation, all these accidents were dissipated, he went with ease to stool, he had no pain and wished to be discharged.

*Second observation.*—About fifteen years ago, a very wealthy banker, about forty-five years of age, of a bilious temperament, consulted the Baron Dupuytren for hæmorrhoids that were constantly in a state of hæmorrhage. These sanguineous discharges had reduced him to a state of great debility and anemia. Pale and debilitated, he visibly emaciated; he was unable to attend in his counting-house; to write a letter was very fatiguing, and almost impossible to him. M. Dupuytren, after examining him, recognized the existence of internal hæmorrhoids, and proposed excision, which was quickly agreed to. Some days after, he proceeded in the following manner:—The patient having taken an enema and a hip bath, lay on the edge of the bed, the thighs separated: violent strainings protruded the hæmorrhoids, which were immediately seized with a forceps with large blades and excised, not without much trouble; no external hæmorrhage manifested itself. M. Dupuytren did not leave the patient; at the end of a quarter of an hour he perceived him become pale, fall into a state of weakness more and more decided, the pulse became small and hard, a cold perspiration covered his body, he felt a sensation of heat in the abdomen, which was continually ascending. From these signs the professor could not doubt that internal hæmorrhage had ensued. He immediately recommended the patient to make expulsatory efforts, and a great quantity of scarcely coagulated blood was discharged; cold injections were useless, the hæmorrhage was not stopped; then a pig's bladder stuffed with *charpie* was introduced; this succeeded completely, but it was not without great difficulty that it could be kept in its place, involuntary expulsatory efforts tended incessantly to displace it, and actually did so several times. This hæmorrhage weakened the patient very much, and would undoubtedly have been fatal if it had not been arrested so promptly; in a short time the cure of the patient was completed.

*Third observation.*—The banker whose case we have just considered, had a brother at Berlin, who had almost the same symptoms; this person heard of his brother's cure, and wrote to M. Dupuytren. After the report of a celebrated surgeon of Berlin who attended him, M. Dupuytren was quite convinced of the existence of similar internal hæmorrhoids, and recommended excision. But the accident that happened to the first brother had suggested to him a means of effectually stopping the hæmorrhage, and consequently, obviating the greatest danger attendant on this operation. He gave written directions, and advised cauterization, with a *cauterie en haricot* if the hæmorrhage manifested itself. The surgeon at Berlin did not follow these directions; immediately after the operation he quitted the patient. Not long after his departure, symptoms of internal hæmorrhage presented themselves; the patient became faint, pale and covered with cold perspiration. One of this young brothers, who had been present at the first operation discovered the cause of the malady, and sent for the surgeon immediately, without being able to find him; time was lost, and the danger was imminent; the younger brother had the presence of mind to introduce the bladder, as he had seen it done, into the anus, and stuff it with lint and succeeded in stopping the hæmorrhage, but the loss of blood was so great that the patient was a long time before he recovered.

*Fourth observation.*—A broker, the father of a numerous family, suffered for some years from internal and external hæmorrhoids, by which he was more and more incommoded; he was in such a state as to be unable to walk more than sixty paces without stopping to lean against a stone stud, to gain a momentary relief from his sufferings. Finding that he was obliged to give up his business, and wishing at any price to preserve the power of providing for the wants of his children, he came to M. Dupuytren, who examined him and perceived a double hæmorrhoidal tumor, being in every way two inches and a half in diameter; blood and pus were discharged frequently, and the schirrhous disorganization appeared very great. M. Dupuytren proposed excision to him. Some fatal cases which recently happened under other surgeons, having made great noise, and this man having heard of them, the proposal made him shudder. M. Dupuytren had great trouble to convince him that cauterization of the vessel was sufficient to obviate all fatal consequences. At length the patient consented to allow the operation; but he wished to go to the Hotel-Dieu, as he would be better watched, and in order at the first appearance the hæmorrhage might be combated. The excision was made, some of the vessels were cauterized, and on the twelfth day the patient was perfectly cured.

*Fifth observation.*—M. Ex——, a Scotchman, a cavalry officer in the service of the King of England, unmarried, about forty years of age, of a sanguine temperament, experienced, for more than three years great sufferings, caused by internal hæmorrhoidal tumours, which protuded on the least attempt to go to stool. As the fatigues of his profession considerably augmented the annoyance, he came to Paris to consult M. Dupuytren. In compliance with his advice went into a *Maison de Sante*, where he was operated on by this celebrated surgeon, in the following manner:—The patient lay on his side and made efforts as if at stool; the upper thigh was raised up by an assistant, the operator seized each tumour with a large indented forceps, and with a very sharp bent scissors, excised them successively. There were three tumors, not very voluminous, and as there was but a trifling effusion of blood, M. Dupuytren thought that cauterization might be dispensed with. An assistant was charged to remain with the patient, who was perfectly calm. About five hours after the excision, all the characteristic symptoms of hæmorrhage in the rectum were manifested; anxiety, rigors, inclination to vomit, cold perspirations, sinking of the pulse, convulsive contractions of the limbs, inexplicable agony, vertigo, syncope; tenesmus increasing the patient went to stool, and the expulsion of a considerable quantity of partly coagulated blood gave him visible relief. A cold enema was administered, as M. Dupuytren had directed in such cases; it was returned immediately, and replaced by another that was longer retained. Nevertheless, at the expiration of about an hour, the symptoms returned with increased intensity; they produced complete collapse. The patient requested a notary would be sent for, and hastened to arrange his affairs, prefering death, which he thought was inevitable, rather than submit to cauterization. Dr. Caillard and Dr. Marx took the responsibility upon themselves; they endeavored to tranquilize him; and it may readily be imagined that it was not very easy to cauterize under these circumstances.—With the aid of a *speculum*, the place from whence the blood flowed was easily found, and the effusion stopped by the application of a bent *cauterie en harricot*, heated to a white heat. The hæmorrhage ceased; the alarming symptoms were dissipated; the inflamation which results from cauterization, and the dysuria that usually accompanies it, yielded very soon to the use of cataplasms, enemata and baths; a wick was kept in the rectum; at the end of a few days the patient was perfectly cured.

*Sixth observation.*—Mr. Joseph Cur——, of Polish origin, a singer at Amsterdam, forty-eight years of age, for several years suffered great pain in expelling the fæcal matter. This difficulty was caused by the presence of internal hæmorrhoids, which altogether was about the size of a hen's egg, During the act of defecation they protuded, and caused a very painful strangulation, which was very difficult to reduce.

These tumours were not accompanied by habitual nor periodical discharges, only when the patient had a constipation; the hardened excrements, occasioned by their pressure, an erosion that gave rise to a slight discharge of blood. Forced by his profession to long standing, and to exertions of the voice that augmented his disease, and his sufferings, he resolved to travel to Paris, in hopes of being cured. M. Dupuytren made him go into a *Maison de Sante*, where two days after he was operated on. The patient lay in a convenient posture; the tumours, three in number, being protuded, were immediately excised. What is remarkable in this case, is, notwithstanding the large volume of the tumours, the excision caused but a slight effusion of blood, and did not require the cautery. A year after his cure we had occasion to see this patient, when there was no failure in the success obtained with an astonishing promptitude. These observations we owe to the complaisance of Dr. Marx; we will in conclusion, add one communicated by our benevolent colleague, Dr. Palliard.

*Seventh observation.*—A man, about forty-seven years of age, of low stature and sanguine temperament, came to the Hotel-Dieu, to be treated for internal and external hæmorrhoids, with which he had been afflicted for fifteen years. These tumours were so painful that he could take but very little exercise, or run for any distance, without causing protusion of the internal hœmorrhoids, which became irritated immediately by the friction of the dress. Repeated inflammation caused a discharge, sometimes sanguine, sometimes purulent, sometimes both one and the other. The act of defecation was a continual torture to the unhappy patient. Should the fear of those accidents that may result from the excision induce us to leave the patient a prey to this disastrous infirmity? The frequent returns of inflammation of the hæmorrhoidal tubercles will bring on disorganization. Besides, it is almost certain that the sanguine and perulent discharges would undermine the patient's constitution, and the sufferings he would undergo would hasten this fatal termination. We did not hesitate; the operation was decided on, for the danger was not inevitable, whilst disorganization would produce certain death. The patient was prepared for the operation by every means likely to insure success. General bleeding was practised, in order to prevent the violence of the inflammation which usually follows the excision of hæmorhoids. A blister was applied to the arm, to prevent the danger which sometimes follows the too sudden suppression of a natural discharge. The patient was kept on low diet, and the day before the operation the intestinal canal was emptied by an aperient. A tumour, composed of seven or eight tubercles, brownish on the outside, but of a brighter color inside, encircled externally the verge of the rectum. When the patient was lying down, without making any effort, all the tubercles were grouped, so as to form a brownish rugged tumour, about the size of a large walnut. When, on the contrary, the patient contracted the abdominal muscles, or strained if at stool, the external tumours opened and exposed a second circular hæmorrhoid, also composed of seven or eight tubercles, but of different color, for they were uniformly of a roseate colour, and covered in all their extent by the internal membrane of the rectum. After having pointed out these different circumstances, M. Dupuytren ordered the patient to lie down on his abdomen, and make efforts as if at stool. This caused the protusion of the internal hæmorrhoids, which were seized with a dissecting forceps, and each of the tubercles that composed it were excised. The same was done with the external tumour, and immediately after, a cautery heated to a white heat, was applied to the bleeding parts of the wound; some hours after, a small wick was introduced into the anus, and afterwards a cerate. He could retain them but a very short time. During the day he complained of transient cholic pains, (diet, diluting drink, &c.) The next day the cholic pains were more violent and more prolonged, the verge of the anus was tumefied and painful, the patient felt difficulty in voiding urine, feverish symptoms, (bled in the arm, diluting drink, &c.) The third, fourth, fifth and sixth days after the operation, the pains decreased, the urine was freely evacuated, and the feverish symptoms disappeared; the appetite returned, and he was allowed some aliments. The seventh day, as the patient had not been at stool from the day of the operation, an ounce of castor oil was administered to him, and some hours after the bowels were opened five or six stools occurred during the day, they were all accompanied by great pain at the anus, nevertheless, after each of them the patient felt considerable relief. The following day he went freely and naturally to stool, the cholic pains became less and less frequent, but the twelfth day they returned with violence, and were followed by diarrhœa, the cause of which was unknown, (mucilaginous drinks, &c.)—The next day he was in the same state, (treacle one ounce.) The fourteenth day the diarrhœa ceased, and with it the cholic pains, (rice gruel.) On the fifteenth day the patient was quite well. He was allowed a moderate portion of food, he was radically cured of the hæmorrhoids, and the anus remained free in whatever posture he placed himself; defecation caused no pain, and he quitted the hospital completely cured.

6. *Treatment of Prolapsus Ani.*—In prolapsus ani, produced by the invagination of the rectum or by the relaxation and projection of the mucous membrane of the rectum, the following treatment is recommended by Mr. Benjamin Phillips, in a communication in the *Medical Gazette* for December last, by which he states that he always effects a radical cure of the disease.

"The reduction having been accomplished, the patient is placed upon a bed; the pelvis is then raised by pillows, so as to make the buttocks the most elevated portion of the trunk; the thighs and the folds of the nates are then so far removed from each other as to enable us with facility to make any application in the vicinity of the anus. A cautery, heated to whiteness, is now placed in the hands of the operator; who makes, according to the gravity of the case, one, two, three or four applications of the cautery to the margin of the anus.

"The exact point to which the application should be made is dependent upon the gravity of the disease; if it be not of long standing nor obstinate, the application should be made to the margin of the anus, but without implicating the mucous membrane; and the length of the eschar should be about half an inch: this should be the uniform length of the eschar.

"If the case be an aggravated one, the application must be made, not only upon the epidermis but also upon the mucous membrane.

"It must be borne in mind that the object of the operation is to produce such an eschar as will be followed by suppuration; for in the process of cicatrization, which follows suppuration, a fibrous tissue is generated, by which the anus will be so effectually contracted as to prevent the possibility of a recurrence of the disease; and it is upon the accomplishment of this effect that the cure almost entirely depends.

"I have found in every case that the stimulous produced by the application of the cautery has occasioned the developement in the submucous cellular, tissue of an irritation capable of producing a firm and permanent adhesion of the mucous membrane.

"In this operation if the iron be properly heated, the pain is inconsiderable. And here it must be recollected that the application upon the living body of an iron heated to whiteness, occasions much less pain than the application of one merely heated to redness, and that the latter occasions much less pain than a gray heat.

"No other dressing than a piece of dry lint is required, and this is retained between the folds of the nates, and removed only when the patient goes to stool; before it is replaced, the anus should be washed with warm water, by which any irritating matter will be removed.— After the performance of the operations, the contractions of the sphincter, which have been excited by the application of the cautery, continue sometimes for three or four days.

"If any pain be felt for a short time after the application of the cautery, fomentations of warm water will generally remove it.

"I shall conclude this communication by detailing the particulars of two cases of this disease which occurred some months since: one in a spare man of sixty-two, the other in a child of three years. In the elder patient, in whom there was no hæmorrhoidal disease, the mucous membrane projected whenever he went to stool, and had done so during very many years. Occasionally difficulty was experienced in reducing it; and in consequence of this, on some occasions it had remained unreduced for many days.

"When I saw him, the prolapsus had existed for nearly four days; there was exhaled from it a fœted sanious discharge, and it was considerably tumefied. The pain which the patient experienced was very severe whenever the tumor came in contact with his linen, or with any other body. Before I could succeed in reducing it, thirty-six leeches were applied, and a constant fomentation with warm water had been employed for some time. After its reduction, the patient was placed in the position I have already described, and the cautery was applied to the anterior and lateral parts of the anus, including about two lines of the mucous membrane. Some pain was experienced for three or four hours; it was relieved by constant fomentation with warm water. The patient did not go to stool for three days after the operation, and when he did, the membrane no longer protuded, neither has it done so to this day. The younger patient had suffered from prolapsus during five months; it occurred every time he went to stool. In this case only one cauterization was made, and that at the distance of a line and half from the mucous membrane.

"The following day when the child went to stool, no prolapsus occurred, neither has it since. The cicatrization was complete on the twentieth day."

8. *Treatment of Cholera by Cold, as Practised by* Dr. Caspar, of Berlin.—Dr. Caspar was first led to employ cold externally in cholera from the advantage derived from it in the treatment of asphyxia from cold. Physicians originally treated such patients, as cholera has since been treated, by the application of heat; but this practice has long since yielded to friction with ice, snow, &c.

Cold affusions had been previously tried at St. Petersburgh, and at Riga, but stimulants had always been given internally at the same time. The result of this method not having answered the hopes which were entertained, it was abandoned. Dr. Caspar does not proceed in this manner. He advises the employment of cold in the boldest manner; and that inert medicines alone should be used in conjunction with this agent. His method is as follows:—the patient is to be placed in an empty bathing-tub, if his skin is dry and flabby; but if on the contrary, it is covered with a slight moisture, or a profuse clammy sweat, the bathing-tub is to be filled with water heated to 27 degrees of Reaumur, (92.75 Fahr.) just up to the navel of the patient.

An assistant on each side supports the patient, so as to keep his back from the sides of the bathing tub; then four or five buckets of iced water are thrown over his head, breast and back. The quantity of water for a child is from one to three buckets. In very severe cases there is no reaction; in cases less severe the patient sighs, and sometimes makes an effort to escape. The slight reaction which takes place in the commencement would astonish those who have not yet employed this theraputic agent. At a more advanced period the reaction becomes more sensible; this symptom is favorable.

Conjointly with these perpendicular affusions others are made in a horizontal direction, dashed from a certain distance, with as much force as possible upon the chest and stomach. Two buckets of very cold water are sufficient for this operation, half the quantity for a child.—The patient in the commencement exhibits but a very slight, and hardly any impression. The cold dashes ought to be administered as promptly as possible, and repeated according to the severity of the disease every two or four hours. Dr. Caspar says that he has often employed twenty of them in the space of two days or two days and a half; ordinarily ten or twelve will suffice.

After the affusion the patient is to be put to bed, and to be covered up to his neck with woollen blankets well warmed. Under these coverings cold compresses are to be applied over the whole of the chest, abdomen and back. It is indispensable to change them as soon as they become warm. The author regards these compresses as the principal agent in his method, because they, according to him, cause a new re-action. In the commencement those who are severely affected are equally insensible to these applications, but a little later the reaction becomes visible. It should be regarded as a favorable sign when the patient complains of the cold. Dr. Caspar does not think that cold compresses ought to be applied to the breasts of women who are suckling.

The head is also to be covered with cold compresses. They should even be resorted to in cases of slight cholera when the effusions are not employed. No other treatment, according to this author, so surely prevents the typhoid fever. Besides he has remarked that typhus succeeds rather milder cholera, than that which shows itself in all its intensity.

Dr. Caspar causes the feet of cholera patients to be enveloped in woolen cloths steeped in warm water. As much as he is opposed to the apparatus for heating the trunk of the body, as vapour baths, &c. yet he advises that bottles filled with boiling water should be applied to the feet. Often he even orders the lower extremities up to the knees to be placed morning and evening in warm water, to which six ounces of sulphuric acid has been added.

The application of cold compresses should be continued night and day until the pulse re-appears, or from small and contracted becomes fuller.

Dr. Caspar also gives his patients cold spring water to drink, or if the patient prefers it, cold beer. He rejects entirely the use of warm drinks. He also administers cold water enemata, and considers them as particularly indicated when there is constipation, and when the movement of a fluid in the intestines can be felt on the application of the hand to the abdomen. It is then that this author orders one or two enemata each day, composed of equal parts of cold water and of vinegar, sometimes with the addition of a spoonful of kitchen salt. These enemata generally act very promptly, provided the patients, as is seen in old men, are not affected with a paralysis of the intestinal canal. Notwithstanding this, it must not be imagined that Dr. Casper employs cold in all cases, and in all the periods of cholera. On the contrary, he modifies it according to the individual cases, and the forms which this disease presents. With this view he indicates three principal varieties of cholera—1st, mild cholera, or that which has been called cholerine. 2d. Asiatic *febrile* cholera, in opposition to that cholera in which there is a complete absence of pulse; but the author himself admits the inaccuracy of this definition. The treatment which he applies to this form is that which we have described previously. He joins to that an emetic, if the patient exhibit signs of gastric embarrassment, but only in that case. 3d. Cholera *sine pulsu* or cholera asphyxia, that which demands at the first onset the bold use of the affusions and cold dashes.

By this treatment re-action is speedily induced. Then Dr. Caspar is accustomed to draw twelve or fourteen ounces of blood, or fewer if the symptoms of local congestion be less marked. The compresses of iced water are continued during this period only upon the head. He prescribes besides, in the greater number of cases, calomel in conjunction with rhubarb: from three to four grains of calomel, with four grains of rhubarb, every hour in the case of adults. Dr. Casper has renounced completely the employment of stimulants even in the typhoid stage, during which he continues the affusions of cold water and compresses to the head.

Although Dr. Casper points out the method of treatment by cold as the most advantageous, he is far from thinking that it will effect a cure in the majority of cases; he admits that the greater number of cholera patients die whatever may be the treatment which is adopted. However, the following are the conclusions which he believes may be drawn from his observations upon the comparative use of cold and other remedial methods in cholera.

1st. The treatment by cold is suited to the nature of this disease, (Dr. Caspar regards cholera as consisting in a paralysis of the cutaneous system,) or if that pathology is not admitted, to all its most marked phenomena.

2d. When applied with circumspection, more patients are cured by it than by any other method.

3d. It produces a surprising amelioration in the most severe forms, and in the most serious periods of cholera, when no other medication has any effect.

4th. It more surely than any other method prevents the consecutive typhus.

5th. It suits itself more easily to the taste and wants of the patients than the stimulating method.

6th. In cases where death is inevitable, it prolongs life as long as possible.

7th. This method is simple, costing very little, and easy of application.

Dr. Casper has added to the exposition of this method, a great many cases which attest its success.

9. *Dr. Hancock on External Stimulants.*—In the London Medical and Surgical Journal, for September 15 and 22, there is a paper on the utility ef external stimulants in internal inflammations, of which we shall here take a brief notice. Dr. H. first makes some observations on the sensibilities of certain internal tissues, as of the mucous membrane of the stomach and bowels, and then proceeds to the consideration of cutaneous stimulation as offering a powerful means of arresting the progress of inflammation, "by deriving the blood and humors from internal congested parts, equalizing the circulation, restoring a mutual balance between the powers of the heart and the external capillaries, and so ultimately obtaining the desired resolution." While Dr. H. acknowledges the utility of blisters, he considers them, in severe cases, as very partial remedies, being too circumscribed in their operation. The more general application of friction to the skin with lime-juice and pepper, or vinegar and common salt, in cases of severe internal inflammation, is proposed by our author. The following extract, containing facts rather than arguments, we shall insert here, as it illustrates Dr. Hancock's views on the subject of external stimulation.

"The bird-pepper (capsicum frutescens) is the kind I have always used, it being more hot and pungent than the other kinds in general. A small handful should be well bruised in a mortar, so as to reduce the seeds and other parts to a fine powder or mass. The powder sold in the shops should not be trusted, it is shamefully sophisticated, like most other articles. Let the former be mixed with an equal quantity of table salt, and add a pint of lime-juice or good vinegar. With this composition the body is to be rubbed entirely over, until it shall produce a sufficient demonstration of excitement and pain.

In a case on Plantation Anna Regina, which I propose to annex to this paper, the patient was so far advanced as to be quite insensible to the stimulus until the second day of the application, and shewed no sensibility till after at least five or six repetitions of the process.

Another instance of the value of frictions with pepper and lime-juice, is that of a boy, George, belonging to Mr. Evans, one amongst several unexpected recoveries from violent inflammation, in which early bleeding had been neglected, or in which it had failed or proved insufficient, although accompanied by blisters and the usual treatment.

Several other instances occurred in my practice, giving further proofs of the efficacy of the pepper frictions in vehement inflammation of the lungs, as two at Exmouth, an African and a Creole, who had each a violent attack of peripneumony about the same time, and in both whom a relapse occurred, or an aggravation of symptoms from exposure to air, by opening the windows of their apartments during bad weather.

The effects of the remedy were most strikingly manifested on the Creole, Billy. My attendance was required at 9 p. m. when I found him laboring under an excessive aggravation of his disorder, with difficult respiration, cough, anxiety, hot dry skin, and small jerking pulse.* The frictions had been used some days previously, but were discontinued, owing to the great and decided relief they had afforded. I stood by, and assisted in performing the operation effectually by smart frictions over the whole body, with a small handful of capsicum, bruised down with some table salt and lime-juice (we used no weights or measures for this composition.) He said it burnt his skin very much, but most readily submitted, however, as he knew, he said, it would help him, as it had done some days before. On the following day he told me he had not slept much from the burning of the skin, but felt quite relieved of the inward pain. His skin was moist, pulse soft, expectoration free, and he experienced no difficulty in breathing. The application was afterwards repeated a few times more moderately. He had no further symptoms of complaint of the chest, except cough and expectoration, which gradually declined; but the severity of the disease had been such as to cause a serous effusion or anasarcous swelling

* Tongue not dry, however, as in the case of Benjamin, at Sparta.

at the feet, and puffed belly, for which we directed bark, wine, and gentle exercise, and he recovered.

Soon after this, the man named Gilbert, also of Exmouth, was taken with the same complaint, (a very severe peripneumony,) but shortly recovered under the same *regimen*, which the sick nurse said caused him to perspire very freely; and indeed I always found it wonderfully to remove the constriction of the skin in cases where bleeding was inadmissible, and when all hope from this, or other means were gone by.

The man Mars, on the same estate, recovered from pneumonia under the same remedy, the year ensuing, and that from an almost hopeless condition. Another case of the same kind, Richard, at Hampton court, rapidly recovered from a severe attack of peripneumony, under the use of the pepper frictions. I was absent, and the hospital attendants commenced it of their own accord, on the second and third day of the disease, as from having seen its benefits in so many previous cases, they considered it a certain remedy. This was stated to me by Mr. M'Lenan, a very careful and excellent young man who superintended the sick-house.

I must not fail to note here a previous case in a Creole boy, called Wellington, on the same estate, in a vehement attack of pleurisy. He came into the Hospital and was bled twice the same day, and was blistered on the next, with a smaller bleeding, and blister repeated, and glysters, nitre, and antimony, from the first, with barley-water, &c. On the third day his respiration was difficult and painful; the fever and all the symptoms were greatly aggravated; his appearance was ghastly, his tongue dry, and he swooned on sitting up in bed. This afternoon I directed frictions with bruised pepper (capsicum frutescens), and the lime-juice.* It was faithfully executed by the sick-nurse, who followed up this idea, had withal bound up his feet, legs, and arms, with the same composition, as a cataplasm. I found him now to my surprise, sitting up and free from complaint, with composed, easy respiration, and no fever and saying he felt no pain. The sick-nurse said he appeared to derive sensible relief at about eleven o'clock the preceding night, the hour in which the fever and other symptoms had been aggravated on each preceding night.

In this case I remarked that the sick-nurse seized the idea, and commenced the process with alacrity, owing to her having witnessed its success in two former similar cases of forlorn hope, in which I had prescribed the pepper friction, and in each of which it was applied over the whole body, and often repeated. I accordingly found powdered pepper now sticking all over the boy's skin. And I was told that he complained of nothing from this time but hunger and the heat of the pepper. Such an early, sudden, and decisive resolution is not

* Lime or lemon juice, being ever at hand, was commonly employed; but good vinegar is fully equivalent, and is free from the unpleasant clamminess resulting from lime-juice and salt. I am told this practice has been verified by two eminent and skilful physicians of Essequebo, Drs. Bell and Buchanan.

to be expected from the use of the common remedies, even with the best efforts of nature.

A still more surprizing recovery was effected last year (1824) by the same means, in the boy called Christmas, at Anna Regina. This lad, (about fifteen years of age,) recovered from the most hopeless state of inflammation which I ever knew, or which it is possible to conceive one to escape from. The patient was in the latter stage of measles, and the inflammation of the pulmonary organs was in this case, so excessive that the patient could scarcely breathe, and was nearly suffocated; had constant fever, dry skin, parched tongue, and such a metastasis on the brain as rendered him delirious and perfectly insensible for about two days. Both the pulse and respiration were nearly extinct. His eye was glassy, and covered with a film; his appearance, in short, was most cadaverous; he was totally unable to swallow, and the *raucum* or rattle in the throat, withal announced a speedy dissolution.

Mr. Gordon, the hospital attendant, observing that I had omitted this patient in the prescription-book, inquired if any attempt should be made to give him any thing, as cold water, his mouth being dry and parched. I told him, that would only add to his distress by impeding respiration, as we had just witnessed, that nothing could possibly be of any avail; but if he pleased he could try the rubbing with pepper and lime-juice. Having seen its effects at Hampton Court, he commenced instantly (having two assistants employed), and diligently repeating it over the whole body. The patient was entirely insensible to its stimulus until many applications had been made, but the following day he began to shew signs of pain, and soon afterwards he spoke, and complained of the smarting *over all his skin.* From this period the fever abated, the tongue became moist, and perspiration ensued. On visiting him the next day, he was sensible, and raised himself in bed, and said he must have some soup and wine, which were instantly brought him; he actually appeared like one risen from the grave. The fever and all other signs of inflammation had vanished; he complained of no pain; coughed, with a copious expectoration, which at first was dark and fœtid. The rubbing was still repeated two or three times a day, and, although reduced almost to a skeleton, his recovery was rapid and perfect. Mr. Frost, the manager, and Mr. Gordon and others, can attest the truth of this extraordinary recovery. It was, however, only by the most diligent and persevering application of the remedy that the patient was saved.

Soon after this, George, a servant of Mr. Evans, in a desperate peripneumony, recovered under the same treatment. Bleeding and various medicines had been used without effect; both the nurse and patient scouted all other remedies, and boldly asserted that nothing but the rubbing saved him. I was called away for some days, but learned on my return, that in this case, as in the others I had witnessed, the free expectoration and perspiration came on, and fever abated, together with freedom of respiration and cessation of pain, in a short time after the frictions were commenced. The patient also soon acquired a remarkable craving for food.

Another most striking case occurred on Sparta estate. The man Benjamin was attacked with pleurisy, or rather peripneumony. He was bled four or five times during the first four days with blisters and the usual remedies. On the fourth evening, finding him much worse, with dry tongue, great pain in the chest, suffocating breathing, and all the symptoms pointing to a speedy termination, the pepper frictions being resolved on as the only hope remaining, were promptly applied and repeated several times during the night. He took nothing but barley-water, with a little nitre and an opiate pill On the morning following he was quite tranquil; the tongue was moist, heat gone, and skin soft and moist. Mr. Campbell saw him well rubbed again yesterday (Oct, 14). He is now out of danger.

I have passed unnoticed many other cases of less importance, or of less severity.

In similar cases, when remedial treatment can be applied early, the lancet should be employed, and the usual auxiliaries as laxatives, antimonials, diluents, blisters, &c. We ought not to fritter the time and life of a patient under the use of single remedies, as when a formidable enemy is within the walls, we should not put forward a force in single file to repel his approach, and thus trifle till he gets possession of the castle. Yet, how common is it in physic to trust, as we may say, to a single picquet guard, until the breach has become irreparable.

In fine, I have found the above practice most effectual and decisive, altogether, I may say, without a parallel, not having been disappointed in a single instance; and it has been to me a source of high gratification to see patients brought round, under its use, from the most desperate conditions, and such as I had ever before considered to be utterly past recovery."

10. *Clinical observations on the Exhibition of Opium in large Doses, in certain Cases of Disease.* By Dr. W. Stokes, *Physician to the Meath Hospital, Dublin.*—In the second number of our Dublin contemporary there is a paper by Dr. Stokes, on the effects of opium in inflammatory diseases, which deserves notice. Various practitioners in hot climates have demonstrated the utility of opium in conjunction with other remedies, in subduing inflammatory affections, as dysentary, hepatitis, and fever; while Hamilton, Armstrong, and many others have done the same in this country. Dr. Armstrong, indeed, and the late Mr. Hayden, proposed opium *alone* in abdominal phlegmasiæ—and Dr. Stokes appears to follow in the same track.

"The first form of disease in which the use of opium appears peculiarly advantageous, may be stated to be that of *Peritonitis occurring under circumstances where bloodletting cannot be employed.* Now, the following are the circumstances under which I have seen this condition of parts to arise:—

1st.—Peritonitis arising from the escape of fæcal matters into the peritoneal cavity, through a perforating ulcer of the intestine.

2nd.—Peritonitis arising from the bursting of an abscess into the serous cavity.

3d.—Peritonitis occurring after the operation of paracentesis in debilitated subjects.

In addition to these cases which I have myself witnessed, we may add, that low typhoid peritonitis, occurring after delivery, as described by Drs. Cusack and Gooch; and the peritonitis which results from rupture of the intestine, induced by external violence."

There can scarcely be a more appalling accident than perforation of the intestine, and escape of its contents, among the bowels, with the rapid and dreadful peritonitis that ensues. As this accident generally supervenes on a wasting disease, as intestinal or typhoid fever, there is nothing to be gained by bleeding, as the disease runs a rapid course, and prostration of strength exists from the very beginning. We have, then, two indications (according to our author) to pursue—first, to support the strength of the patient;—secondly, to prevent the further effusion into the peritoneal cavity, so that nature may have time and opportunity to surround what has already been extravasated, by boundaries of coagulable lymph. Opium in large doses, is the remedy on which our author depends. Two cases are condensed from the fifth volume of the Dublin Hospital Reports, in one of which great relief was obtained, but the advantage was lost by exhibiting an aperient too soon, when all the bad symptoms recurred, and the patient died. The next case was more successful, and our readers will find it quoted in No XXVIII. of this Journal, page 541. There can be little doubt that, in the case alluded to, there was perforation of the intestine, with consequent extravasation of its contents. The power of bearing opium, (which in this case was given to a great extent) without injury to the nervous system, is very remarkable, and is only explicable by the *pain*, on which the anodyne effects of the opium appear to be expended.

Since the occurrence of these cases, our author has used the same remedy in examples of ordinary peritonitis, where bleeding was inadmissible; and he assures us that he has had no reason to change his high opinion of its powers. In one case where death took place, the opium was borne without the slightest inconvenience. In two cases lately occurring in the hospital, the same treatment has been pursued with the most striking benefit. A case is quoted from the Dublin Hospital Reports, of hepatic abscess, in which the practice here recommended was first tried.

"I have now detailed cases illustrative of the utility of opium in large doses in peritonitis, arising from the introduction of fæcal and of purulent matter into the serous cavity. I would further propose it as a remedy in cases of rupture of the bladder and uterus, and in peritonitis after the operation for strangulated hernia. I am at present trying its powers in a case of recent pneumothorax from pulmonary fistula. In two cases of peritonitis after tapping, where the patients were in a low state previous to the operation, the exhibition of these large doses of opium, without drawing, any blood generally or locally, has succeeded in my hands in removing the disease and saving the life of the patient. This appears to be a peculiarly appropriate case for the opium treatment. The patients are generally cachectic, either from original constitution or the disease. They almost all labor under visceral disease and obstruction, and a state of collapse commonly follows the operation. All these circumstances go strongly against our use of the usual antiphlogistic treatment; it was in a case of this kind which occurred in the old Meath Hospital, in the year 1822, that Dr. Graves first ascertained the great importance of opium in this disease. A woman had labored under ascites, for which she was tapped; the operation was followed by the symptoms of peritonitis. When Dr. Graves saw her, she appeared in the last stage of the disease, she had constant vomiting, hippocratic countenance, cold extremities, the belly exquisitely tender, and the pulse 160 in the minute, and nearly imperceptible. The case appearing hopeless, Dr. Graves determined on merely endeavoring to allay the distressing symptom of vomiting, and admininistered a drachm of laudanum. The patient soon after fell asleep, and awoke refreshed, with a more warm surface and fuller pulse. The vomiting had ceased. The same remedy was used in smaller doses every fourth hour, and in the course of two days all the unpleasant symptoms had disappeared.

I now proceed to submit some cases of diseases of mucous membranes, where the use of opium has proved efficacious from having witnessed its utility in cases of inflammatory states of the serous membranes, where the inflammation might be termed, for want of a better name, *asthenic;* it appeared probable, that the same condition of mucous membranes might be benefitted by it.

It is now some months since I was called to see a gentleman laboring under all the symptoms of gastro-enteritis, in a severe form. He had considerable fever; thirst urgent; constant smaking of the lips; respiration hurried, *without disease of the respiratory system;* a red tongue, and a great tenderness of the belly. The usual treatment was pursued, but the disease showed great obstinacy, and after six weeks continuance the situation of the patient appeared hopeless. At this time a violent bronchitis supervened, and so great were the sufferings of the patient from the accumulation of mucus in the trachea, that on three occasions I left him, never expecting to see him again. It was remarkable, that during this attack, the symptoms of abdominal disease greatly subsided. Under a stimulating treatment he recovered from the bronchitis, only to relapse into his former state. The abdominal symptoms now became still more urgent; the belly swelled from tympanitis; the verge of the anus became surrounded by large and irritable hæmorrhoids; there was extraordinary prostration and constant low delirium. Under these circumstances, a diarrhœa supervened, at first slight, but afterwards so severe as to threaten every day death from exhaustion. A great variety of means were tried, but without avail. At this time, when the patient seemed in articulo mortis, I ordered him a grain of opium every hour; this he took regularly for the first twelve hours, *without any inconvenience*, and he experienced some refreshing sleep. Next day, the remedy was continued in the same dose every second hour, and from this time his improvement was rapid; and I rejoice to say, that he is now in the enjoyment of good health."

This was a fair case, Dr. S. observes, for the employment of this *heroic* remedy, since all other means had failed. The next case is one to which he confesses he cannot but look back on with pleasure, was that of a patient who was admitted in February into the Meath Hospital, complaining of sore throat and shooting pain through both ears. His countenance was haggard—voice raucous—body emaciated.

" An extensive and unhealthy-looking ulcer, covered with a whitish matter, was found to occupy the left tonsil, the back of the pharynx, and left side of the uvula. The patient denied having had venereal, but circumstances led us to suspect this; he had, however, been frequently salivated in India, for abdominal disease and fevers. He first felt the soreness of his throat six weeks before admission, which was the time when his vessel made the British Channel. We ordered the patient the sarsaparilla decoction with nitro-muriatic acid, and touched the sore with a strong solution of nitrate of silver, which caustic was changed in some days for the butter of antimony. No good effect was produced by these means; the soreness extended quite round the uvula, which it rapidly destroyed. The breath became fætid; the cough laryngeal; the patient's appearance was still worse than on admission; his nights were sleepless, and he complained much of pain in the head. I now changed the plan of treatment, omitted the sarsaparilla and the lotion, and ordered a gargle of chloride of lime with the internal use of six grains of opium daily, and an increase of his wine. At once the sore began to assume a more healthy appearance; the fætor of breath diminished greatly, and in a few days wholly disappeared. After a short time, in consequence of want of sleep, we increased the dose to eight grains, on which he has been kept since the 20th of February. The sore is now healed, and the whole state of the patient singularly improved."

At the time of writing, there was in the Meath Hospital, a woman, who, for an enteritis, had taken 18 grains of opium in 48 hours. Previous to the exhibition of opium there had been retention of urine, which gave way under the administration of the remedy.

" Hitherto I have alluded to the employment either of simple opium or its tinctures. In Dr. Bardsley's interesting collection of Medical Observations, several cases are detailed, where in affections of the stomach, the acetate of morphia was employed with benefit. I am persuaded that it is a remedy of great power, particularly in chronic cases of dyspepsia, where there is much acidity. I was consulted some months back by a gentleman who has led a very dissipated life, and who for the last ten years has been a martyr to the worst symptoms of dyspepsia. About two years ago, he had a violent attack of hæmatemetis, and latterly the stomach had become so irritable, that it was scarcely possible to find any article of diet to agree with him. His sufferings were dreadful. After trying other means ineffectually, I ordered the 1-8th of a grain of acetate twice a day. He took the remedy three times in the day, with the most perfect relief. The secretion of acid which had been enormous, was suddenly checked, and the patient in two days declared that he had felt better than he had done at any time for the last ten years. His appetite was good, and he foolishly indulged in articles of diet from which he had long abstained. On the fourth day, while in the highest spirits, he became pale; fell, and threw up several pounds of blood. The remedy was of course omitted. In about a fortnight, all the former unpleasant symptoms returned, and his indigestion was as complete as ever. I now ventured on exhibiting the morphia again, but in the doses of the 1-16th of a grain, twice in the day. This diminished dose again produced the same improvement, but in a few days was followed by a return of the hæmatesmetis.

The next case is one of a gentleman who has been for a length of time in the East Indies, and who has become a victim to hepatic disease. He is subject to attacks of pain in the epigastrium, followed by jaundice and fever. These have been treated by leeching, purgatives, and the use of mercury; but during the last attack, his debility was so great, that I did not wish to venture on this treatment. The acetate of morphia was given twice a day. It was commenced on the 26th December, and continued till the 18th February. The greatest improvement has been made in this gentleman's state. The pain has disappeared; there is now no tenderness; the jaundice has subsided, and what is most remarkable, his bowels now act regularly without the assistance of medicine. He has gained flesh, and his whole appearance is singularly improved."

We have now only to append the conclusions to which our author has come relative to the exhibition of opium.

"1st. That in certain cases of inflammation of serous and mucous membranes, where depletion by bloodletting or other antiphlogistic measures are inadmissible, and the system in a state of collapse, the exhibition of opium has a powerful effect in controlling the disease.

2d. That under these circumstances the remedy may be given in very large doses, with great benefit and safety.

3rd. That this effect then is to raise the powers of life, and remove the local disease.

4th. That the poisonous effects of opium are rarely observed in these cases; the collapse and debility of the patient appearing to cause a tolerance of the remedy.

5th. The cases in which the utility of this practice has been ascertained, are as follow:—

Simple peritonitis, in a stage where bleeding cannot be performed. Low puerperal peritonitis. Peritonitis from perforation of the intestine; from the opening of an abscess into the sac; or lastly; after the operation of paracentesis in debilitated subjects. Violent diarrhæa, supervening in an exhausted subject. Phagedenic ulceration of the throat, in a similar individual. And cases of chronic gastritis, and gastro-duodenitis in patients exhausted by the long continuance of the disease.

6th. The cases in which this mode of treatment would be probably useful are—peritonitis from rupture of the bladder, or uterus, traumatic rupture of the intestine, or after the operation for strangulated hernia.

The last observation which I shall make here is, that in most of these cases, particularly in those of diseases of serous membranes, wine was given in conjunction with the opium, and in all, the patients were supported by a lightly nutritious diet."

This paper is certainly interesting, and the subject important. On this account we have laid a full account of it before our readers.—*Medico-Chirurg. Review.*

11. *Dr. Broussais' Opinion on the Use of large Doses of Tartar-Emetic in Pneumonia.*—The employment of this agent in acute inflammation of the lungs is most efficacious, when the patients have been weakened beforehand sufficiently by bleedings. We are more and more satisfied that it acts, not as a specific in counteracting inflammatory excitement, but altogether as a revulsive, or, in other words, that the relief to the lungs is always in proportion to the rapidity and to the quantity of discharges produced, whether by vomiting, purging, sweating, or flow of urine. It is quite a mistaken notion to suppose that the amendment of a patient begins only when the antimonial ceases to have some sensible effect, or, as it is expressed, when a "tolerance" of the drug is induced. When it checks an inflammation, without causing vomiting or purging, we shall find that profuse sweating or diuresis has generally taken place. But should no evacuation at all occur, the antimonial becomes an irritant to the stomach, and gastritis ensues. Dr B. has seen this repeatedly take place, and therefore, dissuades its employment, whenever there are any symptoms of tenderness of the stomach, whether this be primary or consecutive. He narrates the following case. A soldier entered the "Hospital du Val de Grace," having all the symptoms of an acute pneumonia of both lungs, with a hypertrophy of the heart. He was bled largely and repeatedly, but did not experience relief; indeed the symptoms were aggravated, and the antimonial solution was ordered to be taken every hour. It produced copious purging, but the disease advanced rapidly to a fatal termination.

On dissection, the greater part of the lungs was perfectly normal, congestion being visible only in one part. On an examination of the stomach, its mucous coat was found intensely inflamed, and the inflammation was traced to have extended along the whole extent of the duodenum, as far as one-half of the track of the ileum. The inner surface was much softened, and in patches converted into the appearance of a boiled pulp. In short, this patient, says Dr. Broussais, died, not from a pneumonia, but from an intense gastro-enteritis.—*Anal. de la Médéc Physiol.*

*Obs. by Ed.* The above report appears to us to be exceedingly imperfect and ill drawn up, and does not do much credit to the enlightened physician, as no accurate diagnosis of the transference of the malady seems to have been made before death—*Medico-Chirurg. Rev.*

12. *Cholera, Great Variety in the Treatment of, in Paris.*—We have regretted that we have not noted down each and every mode and method which has been recommended in this country; it would form a document at once curious and instructive, at least so far as in shewing us, how the monster has laughed to scorn all the futile attempts to arrest its fury; and how frail and feeble are the grounds on which the practice of medicine, in the hands of many, is founded. No one, we think, will dispute with us, that the only rational hope of successfully treating this, or any other disease, is by a comprehensive and philosophical scrutiny of its physiology or symptoms during life, and of its pathology, or its appearances after death. We must not look to one, or to another of these singly, but group them together, and cautiously endeavor to trace every link of the chain; it is only thus that we can expect to arrive at any rational conjectures as to the causes and essence of the disease; and till then, the treatment must necessarily be empirical, and on the whole, perhaps as inefficacious, nay, even decidedly hurtful, as beneficial. If, in any future period, a method of cure, at once successful and generally applicable, be discovered, we, or our successors, must look back to the records of this disease, with feelings of little respect for the soundness and sagacity of the present race of medical men; for we have all along permitted ourselves to be too much carried along by an exclusive or undue attention to one particular phenomenon, and on that, as on a pivot, we have made our practice turn; but it is never too late to be wise, and our wisdom will be best displayed, by endeavoring to draw some useful lessons from the blundering errors we have hitherto committed. Some idea may be formed of the malapraxis of the French Physicians, from the following report of the different plans of treatment of the cholera in the various Parisian hospitals.

*Hop. Necker.—M. Bricheteau.* Vapour baths, aromatic infusions—general and local bleedings, sinapisms and stimulating embrocations—wine, æther, and cordials—emetics, cold lemonade, iced water, effervescing draughts, ice laid on the epigastrium, opiate, enemas, opium pills by the mouth. In less severe cases, energetic antiphlogistic treatment.

*Hop. Orphelius—M. Blane.*—Bleeding, emetics, effervescing draughts, purgatives, and then astringents, if the purging is obstinate—iced drinks.

*Hop. Piepus—M. Patrix.* Fumigation with chlorine—bleeding, emetics—narcotic decoction—iced lemonade—blisters on each hypochondrium.

*Hop. St. Antoine.—M. Mailly.* Leeches to the anus and epigastrium—venesection, antispasmodic and opiate drinks—stimulating frictions—infusion of peppermint, with acet. ammoniæ.

*Hop. St. Louis—M. Biett.* Subnitras hydrargiri, charcoal, in doses 1-2 drachm every hour.

*M. Lugol.* External warmth—æther, with laudanum and acet. ammoniæ—pills of acetas morphiæ. For drink, strong tea, well sugared, acidulated, and alcoholised! against the vomitings, seltzer water, either alone or with wine.

*M. Gerdy.* General stimulating frictions—blisters along the spine—sinapisms to the arms, legs, and epigastrium—gaseous laudanised potion—pills of camphor.

*M. Jobert.* Sinapisms along the whole extent of all the limbs—laudanum—leeches to the anus. When vomiting is obstinate, seltzer water, or the withdrawal of all drinks.

*Hop. Salpetriere.—M. Piorry.*—General or local bleeding—hot aromatic infusion—Malaga wine or light punch during the collapse—iced water, and when re-action ensues, leeches, poultices, and gum drinks.

*Hop. Pitité.* Fresh lemonade or warm tea—peppermint and laudanum, opiate enemata. M. Andral employs ipecacuan emetics—excitants during the cold stage—local and general bleedings during reaction—opium in small doses.

*M. Lisfranc.* Tea, lemonade, and punch—enemata, with sulphate of quinine—sinapisms and stimulating frictions.

*M. Velpeau.* Sinapisms, opiates, quinine, enemata, to which are added laudanum and camphor.

*M. Bouillaud.* Bleeding, leeches to abdomen, frequently repeated—iced lemonade. In the state of complete collapse, weak coffee, as a drink, and drawing a heated iron along a flannel band, which has been well soaked in equal parts of liquor ammoniæ and spir. tereb. and applied over the whole length of the spine.

*Val de Grace—M. Broussais.* When there is profuse vomiting and purging, the patient should be given ice alone to swallow: when the state of cyanosis ceases, we ought to substitute drinks. No frictions should be employed. General bleeding, or, what is better, numerous leeches, and afterwards hot poultices to the leech-bites—sinapisms—vapor-baths—leeches and iced water to the head.—*Journ. Complement.*

The simple treatment of Broussais coincides nearly with the cold-water practice in this country.—*Medico-Chirurg. Rev.*

